# Flexible iron: disorder in the ironome brings order to protein structure and function

**DOI:** 10.3389/fmolb.2025.1537164

**Published:** 2025-05-30

**Authors:** Vladimir N. Uversky, Gloria C. Ferreira

**Affiliations:** ^1^ Department of Molecular Medicine, Morsani College of Medicine, University of South Florida, Tampa, FL, United States; ^2^ Byrd Alzheimer’s Center and Research Institute, Morsani College of Medicine, University of South Florida, Tampa, FL, United States; ^3^ Department of Chemistry, College of Arts and Sciences, University of South Florida, Tampa, FL, United States; ^4^ Global and Planetary Health, College of Public Health, University of South Florida, Tampa, FL, United States

**Keywords:** iron, iron-binding proteins, iron-sulfur center, intrinsically disordered proteins, proteinprotein interactions, liquid-liquid phase transition

## Abstract

Iron is one of the most abundant elements on earth. The most recognized role of iron in living organisms is its incorporation in the heme-containing protein hemoglobin, which is abundantly found in the red blood cells that facilitate the oxygen transportation throughout the body. In fact, about 70% of organism’s iron is found in hemoglobin. However, besides being essential for oxygen transport and serving as a crucial component of the molecular oxygen-carrying proteins hemoglobin and myoglobin, iron has a wide range of other biological functions. It is involved in numerous metabolic and regulatory processes and therefore is indispensable for almost all living organisms. Since iron enzymes are responsible for most of the redox metallo-catalysts, it is not surprising that 6.5% of all human enzymes are expected to be iron-dependent. Furthermore, iron-binding proteins account for about 2% of the entire proteome. The ironome encompasses heme-binding proteins, proteins binding individual iron ions, and iron–sulfur cluster-binding proteins. Although the structure-function relations of ordered iron-binding proteins are rather well understood, the prevalence and functionality of intrinsic disorder in iron-binding proteins remain to be evaluated. To fill this knowledge gap, in this study, we evaluate the intrinsic disorder of the human ironome. Our analysis revealed that the human ironome contains a noticeable level of functional intrinsic disorder, with most noticeable applications in protein-protein interactions, posttranslational modifications, and liquid-liquid phase separation.

## 1 Introduction

Iron, with atomic number 25 in the Mendeleev’s periodic table of elements, is the second most abundant metal and the fourth most abundant element, by mass, in the Earth’s crust (Sánchez et al., 2017). Iron exists in a wide range of oxidation states, −2 to +7, although +2 and +3 are the most common. Although biological systems typically use iron in only two oxidation states: Fe^2+^ and Fe^3+^, some rare, short-lived intermediates go higher (Fe^4+^/Fe^5+^), but the extreme ends (−2, −1, +6, +7) are not biologically relevant. Table 1 provides some specific examples of the biological contexts or systems, where different oxidation states of iron are observed.

**TABLE 1:** Some specific examples of biological contexts or systems (and of rare or non-biological contexts) where different oxidation states of iron are observed.

Biologically relevant iron oxidation states			
Oxidation state	Biological role	Examples	References
+2 (Fe^2+^); ferrous iron	Electron donor	Cytochromes, hemoglobin, ferredoxins	
		All-ferrous [4Fe-4S] cluster in nitrogenase Fe-protein	Watt and Reddy (1994), Angove et al. (1997), Angove et al. (1998), Solomon et al. (2021)
+3 (Fe^3+^) ferric iron	Electron acceptor, storage	Ferritin, transferrin, cytochromes	
	Electron transfer, catalysis, and sensing within various biological systems	Oxidized state of the [4Fe-4S] clusters in the high-potential iron-sulfur proteins (HiPIPs)	Carter et al. (1972)
+4 (Fe^4+^) ferryl iron	Enzyme intermediate	Cytochrome P450, chloroperoxidase, horseradish peroxidase, and secondary amine mono-oxygenase (Fe^4+^ = O^=^)	Dawson (1988)
		Ascorbate peroxidase (Fe^4+^–OH)	Kwon et al. (2016)
		Bovine cytochrome *c* oxidase	Weng and Baker (1991), Proshlyakov et al. (1994), Fabian and Palmer (1995), Kitagawa and Ogura (1998)
		Cytochrome *bo* from *Escherichia coli*	Watmough et al. (1994)
	Oxidative reactions	Non-heme Fe^4+^ = O intermediates in α-ketoglutarate-dependent oxygenases	Krebs et al. (2007)
	Electron donor during nitrogen fixation	[Fe-4S] clusters in nitrogenase Fe-proteins	Wenke et al. (2019)
+5 (Fe^5+^) perferryl iron	Hypothetical enzyme intermediate	Proposed in heme enzymes (with porphyrin radical)	
		Nitrido iron(V) porphyrin species	Ghosh (2001), Terner et al. (2001), Watanabe (2001), Weiss et al. (2001)
	Control of the complex reactivity	Mononuclear non-heme nitride iron(V) complexes	Meyer et al. (1999), Grapperhaus et al. (2000)
	Effect on structural, electronic, and magnetic properties	Double perovskites materials with a specific crystalline structure (e.g., La_2_-_*x*_Ca_*x*_LiFeO_6_-_0.5x_)	Goto et al. (2025)

Rare or Non-biological Contexts			
Oxidation State	Context	Notes	References
0 (Fe^0^) elemental iron	Anaerobic bacteria, geology	Used by *Geobacter*, found in meteorites	Lovley and Phillips (1988)
+6 (Fe^6+^)	Prebiotic chemistry, synthetic	Found in ferrate (FeO_4_^2-^), not in living cells	Sharma (2002)
−2, −1, +1, +7	Not relevant	Chemically unstable or unknown for iron in biology	

Iron maintains an oxidation state of Fe^3+^ even in the Earth’s mantle at approximately 500 km depth (Kiseeva et al., 2018). Elemental iron occurs in low oxygen environments, such as deep-sea hydrothermal vents (Waeles et al., 2017; Dick, 2019). Among the iron-related microorganisms identified in deep-sea hydrothermal vents are magnetotactic bacteria (Goswami et al., 2022; Zhong et al., 2022; Nakano et al., 2023). Not only are their magnetotactic iron metabolism and iron sequestration thought to have greatly impacted the iron biogeochemical cycle (Amor et al., 2020; Goswami et al., 2022), but, more fundamentally, iron may have facilitated crucial prebiotic chemical reactions and be at the crux of the origin of life (Russell and Hall, 1997).

In 1988, Wächtershäuser hypothesized that iron and sulfur on mineral surfaces facilitated organic molecule formation (Wächtershäuser, 1988). Shortly after, Wächtershäuser’s “*theory of surface metabolism*” was extended to the modular and interconvertible nature of iron-sulfur clusters and their versatile and essential roles for life (Beinert et al., 1997). In a prebiotic world during early Earth (around 4 Ga or 4,000 million years ago), reduced, methylated sulfur and nitrogen compounds were formed in deep-sea hydrothermal vents or transported to Earth by carbonaceous meteorites (Ernst et al., 2023). Ernst *et al.* proposed that these compounds were demethylated - to methane (CH_4_) and ethane (C_2_H_6_) - in Fenton reactions driven by Fe^2+^ and reactive oxygen species (ROS) generated by photolysis and thermolysis or radiolysis (Ernst et al., 2023). The investigators further advanced the hypothesis that the abiotic CH_4_ and C_2_H_6_ formation could have significantly influenced the chemical evolution of Earth’s atmosphere before the origin of life by not only maintaining warm temperatures but also providing a hydrocarbon substrate for early metabolic processes. Notably, iron-sulfur clusters are considered to be at the origin of life due to their fundamental functions in catalysis, electron transfer, and the synthesis of essential biochemical compounds (Beinert, 2000). Their prevalence in ancient and extant biological systems supports their vital role in the emergence of life (Sourice et al., 2024). While interpretation of early biogeochemical processes may still be debatable, the acceptance of iron-centered chemistry at the origin of life seems indisputable.

The “Great Oxygenation Event”, around 2.4 Ga (Pellerin et al., 2024), marked a dramatic increase in the Earth’s atmospheric oxygen levels, mainly resulting from the photosynthetic activity of cyanobacteria (Falkowski et al., 2008). This event supported the evolution of aerobic respiration and the emergence of more complex life forms, including eukaryotes (Falkowski et al., 2008). Iron chemistry is at the core of the evolutionary shift from anaerobic to aerobic life. Nonetheless, organisms had to develop mechanisms to cope with the obstacles owing to the reactivity of Fe^2+^ with oxygen, which yielded insoluble Fe^3+^ hydroxides, toxic hydroxyl radicals or ROS. Many of the strategic mechanisms relied on the iron coordination and the adopted geometric arrangements of the iron-binding centers in proteins (Pozzi et al., 2015; Olson, 2022; Yao et al., 2024). To mention a few, the iron center is coordinated by four nitrogen atoms from the porphyrin ring and one or two axial ligands (*e.g.*, histidine and cysteine) in heme proteins, such as hemoglobin, myoglobin, and cytochrome P_450_ (Albertolle et al., 2019; Olson, 2022), and non-heme iron proteins utilize oxygen, nitrogen, and sulfur ligands from amino acid residues. The wide chemical gamut of ligand atoms that surround the iron atom provides the functional diversity of iron-containing proteins (Pozzi et al., 2015; Albertolle et al., 2019; Olson, 2022; Yao et al., 2024).

In an earlier study, Andreini et al. (2018) represented the human iron proteome (human ironome), such that human iron-binding proteins were grouped based on the chemical nature of their metal-containing cofactors: Individual iron ions, heme cofactors, and iron–sulfur clusters (Andreini et al., 2018). These authors revealed that the human ironome consists of about 400 proteins, thereby accounting for approximately 2% of the entire human proteome (Andreini et al., 2018). In the ironome, there are 35%, 48%, and 17% of proteins binding individual iron ions, heme-binding proteins, and iron–sulfur cluster proteins, respectively. The authors also pointed out that more than half of the human ironome is represented by enzymes, with 6.5% of all human enzymes being predicted to be iron-dependent (Andreini et al., 2018). Finally, the authors emphasized that the human iron-binding proteins are unevenly distributed among the various cellular compartments, being especially enriched in the mitochondrion and the endoplasmic reticulum, and that malfunction of these proteins is frequently associated with various pathologies (Andreini et al., 2018).

Although the architecture of iron centers has been extensively investigated in relation to their function in well-structured proteins, the assessment of the abundance, distribution and relevance of iron coordination and iron-binding centers in intrinsically disordered proteins remains to be undertaken. In this study, we evaluate the intrinsic disorder of the human ironome. To this end, we used several bioinformatics tools, such as RIDAO (Dayhoff and Uversky, 2022), STRING (Szklarczyk et al., 2011), FuzDrop (Hardenberg et al., 2020; Hatos et al., 2022; Vendruscolo and Fuxreiter, 2022), D^2^P^2^ (Oates et al., 2013), and AlphaFold (Jumper et al., 2021; Varadi et al., 2024) for comprehensive analysis of the intrinsic disorder propensity of these proteins and the roles of disorder in their functionality. Our results indicated that the members of human ironome contain noticeable levels of functional intrinsic disorder. This presence of protein intrinsic disorder contributes to protein-protein interactions, posttranslational modifications, and liquid-liquid phase separation among the human ironome members.

## 2 Materials and methods

### 2.1 Dataset assembly

In this study, we analyzed the human ironome described in (Andreini et al., 2018), where the human iron-binding proteins were grouped into three categories: 138 proteins binding individual iron ions, 190 heme-binding proteins, and 70 iron–sulfur proteins. The sequence IDs corresponding to these three subironomes were retrieved from the Supplementary Tables S1–S3 reported in (Andreini et al., 2018). These IDs then were used to retrieve the protein amino acid sequences (in FASTA format) from UniProt database (UniProt_Consortium, 2024). Resulting datasets of the iron ion-, heme-, and iron-sulfur cluster-binding proteins included 138, 190, and 70 members, respectively. The retrieved sequences were used in the various bioinformatics analyses. As a comparison, we used 5,066 human calcium-binding proteins found in UniProt.

### 2.2 Intrinsic disorder analysis

Intrinsic disorder predispositions of all human iron-binding proteins were determined using a set of commonly used per-residue disorder predictors including PONDR® VLS2, PONDR® VL3, PONDR® VLXT, PONDR® FIT, IUPred-Long, IUPred-Short (Romero et al., 2001; Obradovic et al., 2005; Peng et al., 2005; Peng et al., 2006; Xue et al., 2010; Meszaros et al., 2018), outputs of which were assembled using the Rapid Intrinsic Disorder Analysis Online (RIDAO) platform that allows analyzing individual proteins and protein sets (Dayhoff and Uversky, 2022). The proteins were classified based on their level of disorder using their percent of predicted intrinsically disorder residues (PPIDR), i.e., residues exceeding the 0.5 threshold. In this classification, proteins are considered as ordered, moderately disordered, or highly disordered if their PPIDR values are less than 10%, between 10% and 30%, and greater than 30%, respectively (Rajagopalan et al., 2011; Uversky, 2020). We also used mean disorder scores (MDS) calculated as a protein length-normalized sum of all the per-residue disorder scores to classify proteins as highly ordered, moderately disordered or flexible, and highly disordered, if their corresponding MDS values were MDS <0.15, 0.15 ≤ MDS <0.5, MDS ≥0.5.

### 2.3 CH-CDF analysis

Combining the outputs of the two binary disorder predictors, charge-hydropathy (CH) plot (Uversky et al., 2000; Oldfield et al., 2005) and cumulative distribution function (CDF) plot (Oldfield et al., 2005), generates grounds of the ΔCH-ΔCDF analysis (Mohan et al., 2008; Sun et al., 2011; Xue et al., 2012) that provides additional means for the classification of disorder status of query proteins (Huang et al., 2012). Here, for each query protein, the sequence-specific ΔCH and ΔCDF values are calculated as the vertical distance of the corresponding point in CH-plot from the boundary (ΔCH) or the average distance between the order-disorder boundary and the corresponding CDF curve ΔCDF, respectively (Mohan et al., 2008; Sun et al., 2011; Huang et al., 2012; Xue et al., 2012). Then, a CH-CDF plot is generated by plotting ΔCH against ΔCDF. In the CH-CDF phase space, the proteins in the bottom-right quadrant are predicted to be ordered by both predictors and therefore are classified as ordered/compact, the proteins in the bottom-left are predicted to be ordered by CH and disordered by CDF [they are classified as native molten globules or hybrid proteins containing sizable ordered domains and intrinsically disordered regions (IDRs)], the proteins in the top-left quadrant are predicted to be disordered by both CH and CDF (they are classified as native coils or native pre-molten globules), whereas the proteins in the top-right are predicted to be disordered by CH and ordered by CDF, and (Mohan et al., 2008; Sun et al., 2011; Huang et al., 2012; Xue et al., 2012). Data for CH-CDF analysis were generated using RIDAO (Dayhoff and Uversky, 2022).

### 2.4 Disorder-based functional annotations

For a set of the most disordered iron ion-, heme-, and iron-sulfur center-binding proteins, we conducted additional analysis of potential disorder-based functionality. To this end, we used the Database of Disordered Protein Predictions (D^2^P^2^) showing the present and localization of the disorder-based binding sites (based on the outputs of the ANCHOR algorithm) and sites of various posttranslational modifications (PTMs) (Oates et al., 2013). D^2^P^2^ also gives Structural Classification of Proteins (SCOP) domain predictions based on the SUPERFAMILY predictor and disorder predictions based on PONDR VLXT, PONDR VSL2b, PrDOS, PV2, ESpritz-DisProt, Espritz-XRay, Espritz-NMR, IUPred-Long, and IUPred-Short predictors (Oates et al., 2013).

### 2.5 Protein-protein interaction networks

To analyze the interactivity of all human iron-binding proteins, we utilized the Search Tool for Recurring Instances of Neighboring Genes (STRING) platform (http://string-db.org/) (Szklarczyk et al., 2011) using the medium confidence of 0.4 for the minimum required interaction score. STRING generates PPI networks using several pieces of evidence, such as known interactions from curated databases and experimentally determined, predicted interactions from gene neighborhood, gene fusions, and gene co-occurrence, as well as other sources, such as text-mining, co-expression, and protein homology. In our analyses, we selected an option to show network edges based on their confidence, where the line thickness indicates the strength of data support. We also used STRING to check the individual interactability of the most disordered iron ion-, heme-, and iron-sulfur cluster-binding proteins.

### 2.6 Liquid-liquid phase separation propensity

The propensity of the members of human ironome for spontaneous liquid-liquid phase separation (LLPS) was evaluated using the FuzDrop platform (Hardenberg et al., 2020; Hatos et al., 2022; Vendruscolo and Fuxreiter, 2022). FuzDrop evaluates the probability of a given protein to spontaneously form a droplet state through LLPS (p_LLPS_). Resulting data can be used to classify proteins as droplet-drivers, which are protein with p_LLPS_ ≥ 0.60 that can spontaneously undergo liquid-liquid phase separation, or droplet-client proteins, which have p_LLPS_ < 0.60 but possess droplet-promoting regions (DPRs, i.e., regions that contain at least five consecutive residues with the residue-based droplet-promoting probabilities p_DP_ ≥ 0.60), which can induce their partitioning into condensates (Hardenberg et al., 2020; Hatos et al., 2022; Vendruscolo and Fuxreiter, 2022).

### 2.7 Structure modeling using AlphaFold

For a set of the most disordered representatives of human heme- (UniProt IDs: O15534 and O14867), iron ion- (UniProt IDs: O43151 and O15054), and iron-sulfur cluster-binding proteins (UniProt IDs: Q6FI81 and O60673), their 3D structures were modeled using AlphaFold, which is an AI-based computational tool that predicts the 3D structure of a protein given its amino acid sequence (Jumper et al., 2021). Corresponding structural models were retrieved from the AlphaFold Protein Structure Database (https://alphafold.ebi.ac.uk/), which provides structure coverage for over 214 million protein sequences (Varadi et al., 2024).

## 3 Results and discussion

**FIGURE 1:**
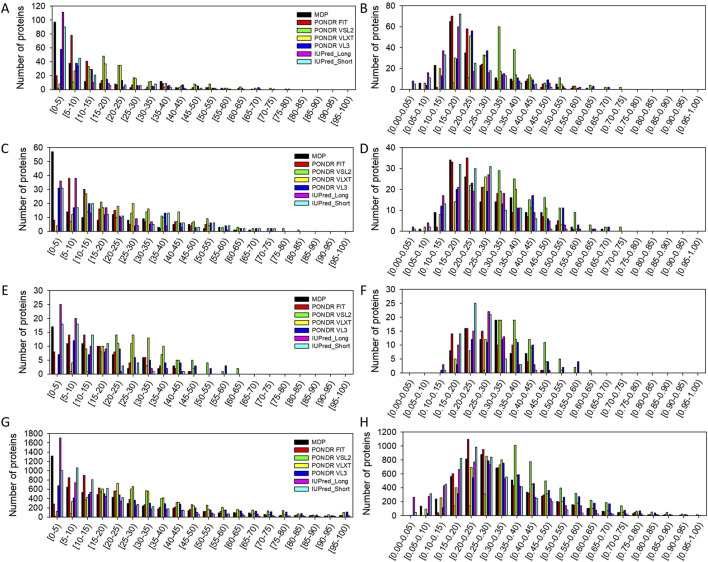
Distribution of the human heme-binding proteins **(A and B)**, iron ion-binding proteins **(C and D)**, iron-sulfur cluster-binding proteins **(E and F)**, and the human calcium-binding **(G and H)** proteins based on their PPIDR **(A, C, E, and G)** and MDS **(B, D, F, and H)** values evaluated by various per-residue disorder predictors utilized in this study.

First, we evaluated the overall intrinsic disorder predisposition of the members of the human ironome. To this end, we examined the distribution of these proteins based on their PPIDR and MDS values. This analysis indicated that the human heme- (Figures 1A,B), iron ion- (Figures 1C,D), and iron-sulfur cluster-binding proteins (Figures 1E,F) are characterized by noticeable levels of predicted intrinsic disorder, as evidenced by several per-residue disorder predictors used in this study. The PPIDR and MDS distributions in the human ironome are not too different from the analogous distributions calculated for the much larger set of human calcium-binding proteins (see Figures 1G,H). However, this analysis revealed that there are still some differences between the ironome and calceome. For example, although the members of the ironome and the calceome tend to have short IDRs (shorter than 30 residues), IDRs longer than 60 residues are almost completely absent in the ironome, whereas a noticeable fraction of the calceome was predicted to have IDRs with the length exceeding 60 consecutive residues (e.g., as per the PONDR® VSL2 analysis, 863 (17.0%) calcium-binding proteins have such long IDRs, whereas based on the MDP outputs (which is the more conservative predictor), human calceome includes 411 (8.1%) proteins with long IDRs). Even though the ironome has a limited number of proteins with very long IDRs, MDS distributions of the heme-, iron ion-, and iron-sulfur center-binding proteins are not too different from that of the calceome.

**FIGURE 2:**
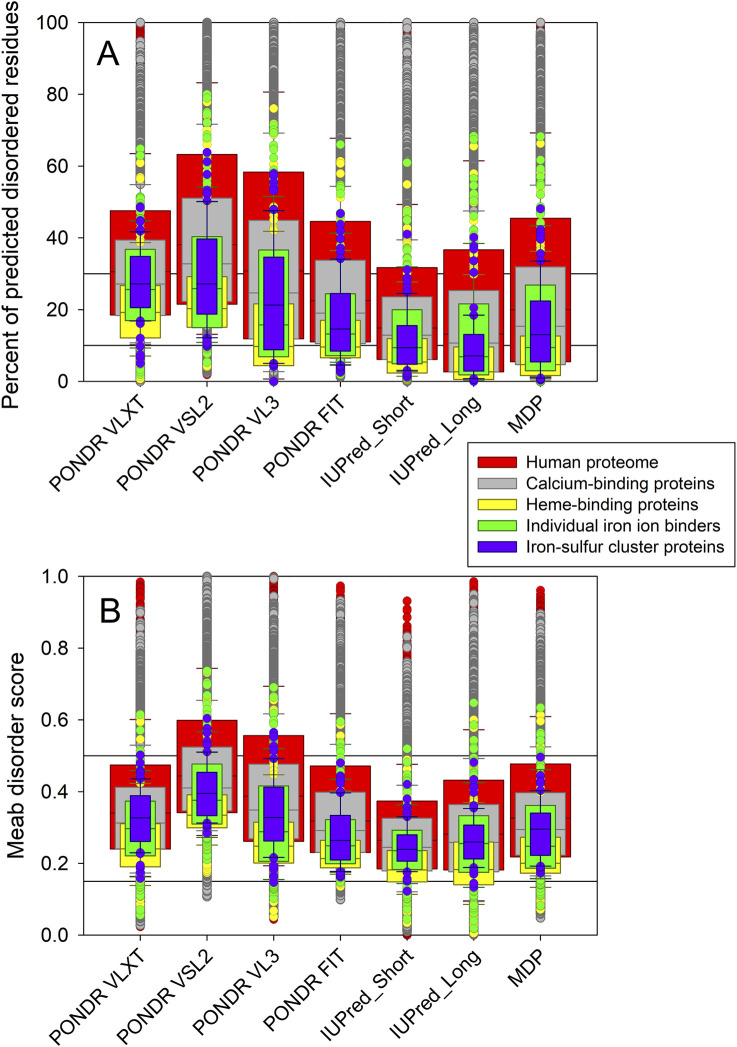
Nested box-plots comparing the overall disorder predispositions of human heme- (yellow bars), iron ion- (green bars), and iron-sulfur cluster-binding proteins (violet bars) with human calceome (gray bars) and entire human proteome (red bars) evaluated by their PPIDR **(A)** and MDS **(B)** values derived based on the outputs of six commonly used per-residue disorder predictors, PONDR® VLXT, PONDR® VSL2, PONDR® VL3, PONDR® FIT, IUPred_Short, and IUPred-Long, as well as mean disorder prediction (MDP) calculated as an average of outputs of these six predictors.

Figure 2 offers a comparative view of the overall intrinsic disorder propensities across human heme-, iron ion-, and iron-sulfur cluster-binding proteins, as well as the human calceome and the entire human proteome. This comparison is presented through nested box plots summarizing the outputs of the disorder predictors used in this study. Figure 2A displays box plots based on the predicted percentage of intrinsically disordered residue (PPIDR) values, while Figure 2B shows box plots based on the corresponding mean disorder score (MDS) values. These analyses reveal that both the calceome and the ironome are, on average, less disordered than the full human proteome. Among the ironome members, iron-sulfur cluster-containing proteins exhibit the highest disorder levels, whereas heme-binding proteins are predicted to be the most ordered.

**FIGURE 3:**
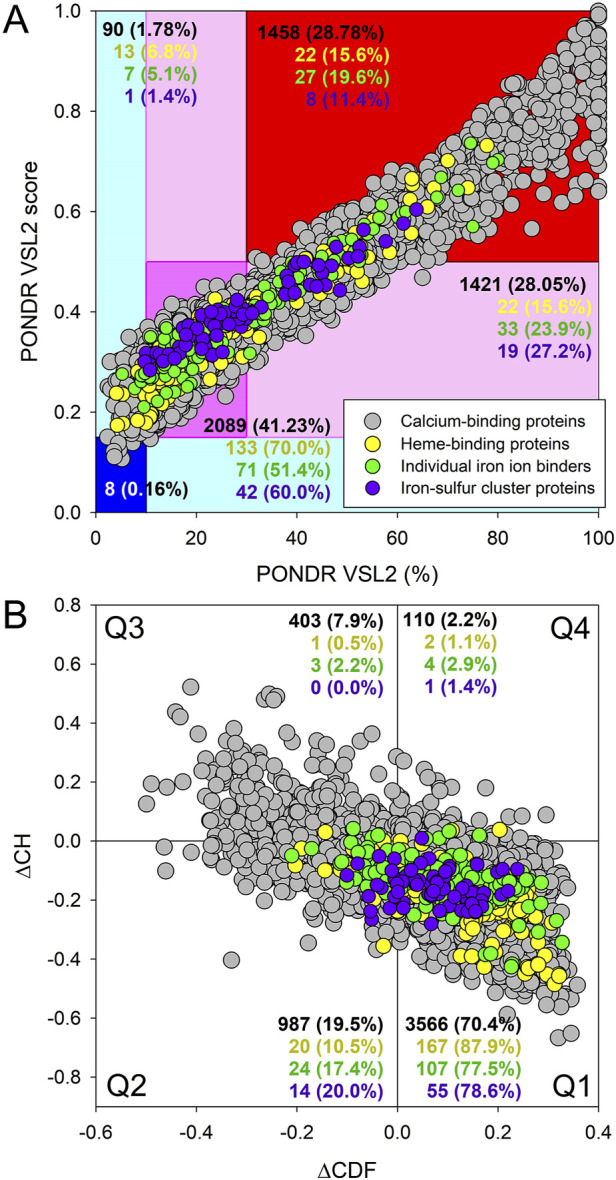
Evaluation of the global disorder status of the human ironome (data for heme-, iron ion-, and iron-sulfur cluster-binding proteins are shown by yellow, green and purple colors, respectively) in comparison with the calceome (gray circles). **(A)** PONDR® VSL2 score vs PONDR® VSL2 (%) plot. Here, each point corresponds to a query protein, coordinates of which are evaluated from the corresponding PONDR® VSL2 data as its mean disorder score (MDS) and percent of the predicted intrinsically disordered residues (PPIDR). Color blocks are used to visualize proteins based on the accepted classification, with red, pink/light pink, and blue/light blue regions containing highly disordered, moderately disordered, and ordered proteins, respectively (see the text). Dark blue or pink regions correspond to the regions, where PPIDR agrees with MDS, whereas areas in which only one of these criteria applies are shown by light blue or light pink. **(B)** CH-CDF plot, where coordinates for a protein are calculated as the average distance of its CDF curve from the CDF boundary (X-axis) and its distance from the CH boundary. Protein classification is based on the quadrant, where the proteins are located: Q1, proteins predicted to be ordered by both predictors; Q2, proteins predicted to be ordered to by CH-plot and disordered by CDF; Q3, proteins predicted to be disordered by both predictors; and Q4, proteins predicted to be disordered by CH-plot and ordered by CDF. [Color code is the same as in panel **A**].

The idea that the members of the ironome and calceome contain noticeable, but different, levels of disorder is further supported by Figure 3 showing the classification of the disorder status of these proteins based on the outputs of the per-residue disorder predictor PONDR® VSL2 (Figure 3A) or the ΔCH-ΔCDF analysis (Figure 3B). In fact, based on the accepted classifications, where the proteins with the PPIDR <10%, 10% ≤ PPIDR <30%, and PPIDR ≥30% values are considered as ordered/mostly ordered, moderately disordered, and highly disordered (Rajagopalan et al., 2011; Uversky, 2020) and as ordered, moderately disordered or flexible, and highly disordered, if their MDS <0.15, 0.15 ≤ MDS <0.5, MDS ≥0.5. Further, none of the iron-binding proteins was predicted as ordered by both MDS and PPIDR, and none of those proteins was predicted as mostly ordered based on their MDS values, with 6.8%, 5.1%, and 1.4% of heme-, iron ion- and iron-sulfur cluster-binding proteins being predicted as mostly ordered based on their PPIDR values, with the remaining iron-binding proteins being either moderately or highly disordered. On the other hand, Figure 3A shows that eight calcium-binding proteins (0.16%) were predicted as ordered by both MDS and PPIDR, and 98 members of the calceome (1.9%) were predicted as mostly ordered based on their PPIDR values. These numbers do not differ much from the corresponding values reported earlier for the entire human proteome, where 0.4% and 5.1% of proteins are predicted as ordered by both MDS and PPIDR and as mostly ordered based on their PPIDR values, respectively (Mohammed and Uversky, 2022). Figure 3A also shows that the ironome and calceome possess noticeable differences in the peculiarities of their disorder distribution. In fact, calceome contains significantly more highly disordered proteins predicted by both MDS and PPIDR than any of the subironomes, which are predicted by both MDS and PPIDR to have very high contents of moderately disordered proteins (see the corresponding protein contents of the dark pink region in the MDS vs. PPIDR plot). Figure 3B provides further support to the important notion that the ironome in general is less disordered than the calceome, with proteins interacting with heme-binding proteins being the most ordered among all the datasets analyzed in this study.

These conclusions are further confirmed by the results of the Kruskal–Wallis one way analysis of variance (ANOVA) on ranks of these datasets using the PPIDR and MDS outputs of PONDR® VSL2 (PPIDR_VSL2_ and MDS_VSL2_, respectively). This analysis revealed that all the differences in the median values among the groups were greater than would be expected by chance, showing a statistically significant difference (P = <0.001). Based on their median PPIDR_VSL2_ values, the analyzed datasets can be arranged as follows: entire proteome (38.11% ± 0.23%) > calceome (32.72% ± 0.38%) > iron-sulfur cluster-binding proteins (27.15% ± 2.06%) > iron ion-binding proteins (25.85% ± 1.88%) > heme-binding proteins (20.28% ± 1.36%). Similarly, based on their median MDS_VSL2_ values, the analyzed datasets are ranged as: entire proteome (0.4432 ± 0.0015) > calceome (0.4102 ± 0.0026) > iron-sulfur cluster-binding proteins (0.3950 ± 0.0115) > iron ion-binding proteins (0.3756 ± 0.0121) > heme-binding proteins (0.3345 ± 0.0093). Similar ranking was observed using the more stringent predictor, MDP. Specifically, based on their median PPIDR_MDP_ values, the analyzed datasets ranked as follows: were ranged entire proteome (20.00% ± 0.23%) > calceome (15.35% ± 0.38%) > iron-sulfur cluster-binding proteins (12.36% ± 1.85%) > iron ion-binding proteins (9.46% ± 1.79%) > heme-binding proteins (4.65% ± 1.27%). When based on their median MDS_MDP_ values, the ranking of the analyzed datasets was: entire proteome (0.3260 ± 0.0015) > iron-sulfur cluster-binding proteins (0.2954 ± 0.0115) > calceome (0.2940 ± 0.0025) > iron ion-binding proteins (0.2477 ± 0.0124) > heme-binding proteins (0.2005 ± 0.0094).

Repeating these analyses for the human proteome, the human caleome, and the entire human ironome—which dataset that combines heme-binding, iron ion-binding, and iron-sulfur cluster-binding proteins—consistently confirmed that the ironome exhibits the lowest level of intrinsic disorder. Specifically, the ironome showed a median PPIDR_VSL2_ of 22.21% ± 1.00%, a median MDS_VSL2_ of 0.3542 ± 0.0065, a median PPIDR_MDP_ of 6.97% ± 0.94% and a median MDS_MDP_ of 0.2340 ± 0.0067. All differences observed among the groups were statistically significant.

Altogether, the results gathered here indicated that the human ironome has a noticeable level of disorder, being, however, significantly less disordered than the human calceome. These observations can be rationalized from the major differences in how organisms handle calcium and iron, since a wide range of human vascular and other progressive inflammatory and degenerative diseases is associated with dysregulation of iron metabolism (Kell, 2009). As it was above discussed, to cope with the obstacles owing to the reactivity of Fe^2+^ with oxygen, which yields insoluble Fe^3+^ hydroxide making iron unavailable, or toxic ROS causing cellular damage, organisms had to develop special strategic mechanisms relying on the precisely controlled regulation of iron metabolism (Chifman et al., 2014) combined with sequestering iron inside specific protein structures to avoid the undesired release of free iron. Placing reactive iron (Fe^2+^) inside iron-binding proteins, typically located within specific iron-binding centers characterized by rigid geometric arrangements and tightly controlled coordination, helps preventing unwanted iron reactivity and potential toxicity.

The different handling of calcium ions in comparison with ferrous and ferric iron is further reflected in their binding constants to proteins. Binding constants of Ca^2+^ to proteins span from nanomolar to millimolar, with this variability required for a wide range of cellular responses to changes in calcium concentration (Andre and Linse, 2002). Similarly, ferrous binding metalloproteins is characterized by iron dissociation constants (*K*_*d*_) falling within the micromolar to nanomolar range (Cook et al., 2006; Kondapalli et al., 2008; Shi et al., 2008). However, binding of ferric iron can be much tighter than that of ferrous iron, as illustrated by transferrin that binds Fe^3+^ with a *K*_*d*_ value in the order of 10^–22^ M, but it does not bind ferrous iron (Martin et al., 1987). In addition, the binding affinity of ferric binding protein, a bacterial transferrin, for Fe^3+^ can be anion dependent as illustrated by the effect of synergistic anions (arsenate, citrate, NTA, ozalatephosphate, and pyrophosphate) on the effective Fe^3+^ binding constants (*K′*_*eff*_) of bacterial (*Neisseria*) transferrin, which range from 1 × 10^17^ M^−1^ to 4 × 10^18^ M^−1^ (Dhungana et al., 2003).

The iron-binding centers in proteins are commonly characterized by rigid geometric arrangements that ensure the required tight iron coordination. Iron can be directly coordinated by His, Cys, Asp, and Glu residues, with other amino acids, such as Tyr, Lys, and Met being also able to interact with iron in specific contexts (Baker et al., 2003; Weber et al., 2021). Similarly, heme is typically coordinated in proteins by His, Cys, Met, Tyr, and Lys residues (Li et al., 2011). Furthermore, it was observed that heme binding pockets are enriched in aromatic and non-polar amino acids, and relatively depleted of charged residues, such as Glu, Asp, and Lys (Li et al., 2011). Although the primary amino acids responsible for coordination of the iron-sulfur clusters in proteins are Cys residues that bind iron-sulfur clusters through iron ions (with the CX_3_CX_2_C motif being commonly found within the cluster binding pocket), some other residues, such as His, Asp, and Glu can be sometimes involved in cluster ligation (Tong et al., 2022; Vallieres et al., 2024). In contrast, calcium ions in calcium-binding proteins, are primarily coordinated by amino acid residues with oxygen-containing side chains, such as Asp, Glu, Asn, Gln, Ser, Thr, and Tyr (Kretsinger, 1976; McPhalen et al., 1991). Amino acid residues can be classified based on their prevalence in proteins as either order-promoting—more commonly found in structured, ordered proteins—or disorder-promoting—more frequently occurring in intrinsically disordered proteins or regions). Order-promoting residues include Cys, Trp, Tyr, Ile, Phe, Val, Leu, His, Thr, and Asn, while disorder-promoting residues include Ala, Gly, Asp, Met, Lys, Arg, Ser, Gln, Glu, and Pro (Dunker et al., 2001; Romero et al., 2001; Williams et al., 2001; Radivojac et al., 2007; Vacic et al., 2007). According to this classification, many residues involved in coordinating iron, iron-sulfur cluster, and heme tend to be order-promoting, whereas calcium coordination is predominantly mediated by disorder-promoting residues. These differences in amino acid composition likely influence the structural disorder propensities of the respective proteins. It can also be hypothesized that the distinct disorder predispositions obxerved in the ironome and calceome reflect their differing functional specializations.

### 3.1 Protein-protein interaction network of the human ironome

**FIGURE 4:**
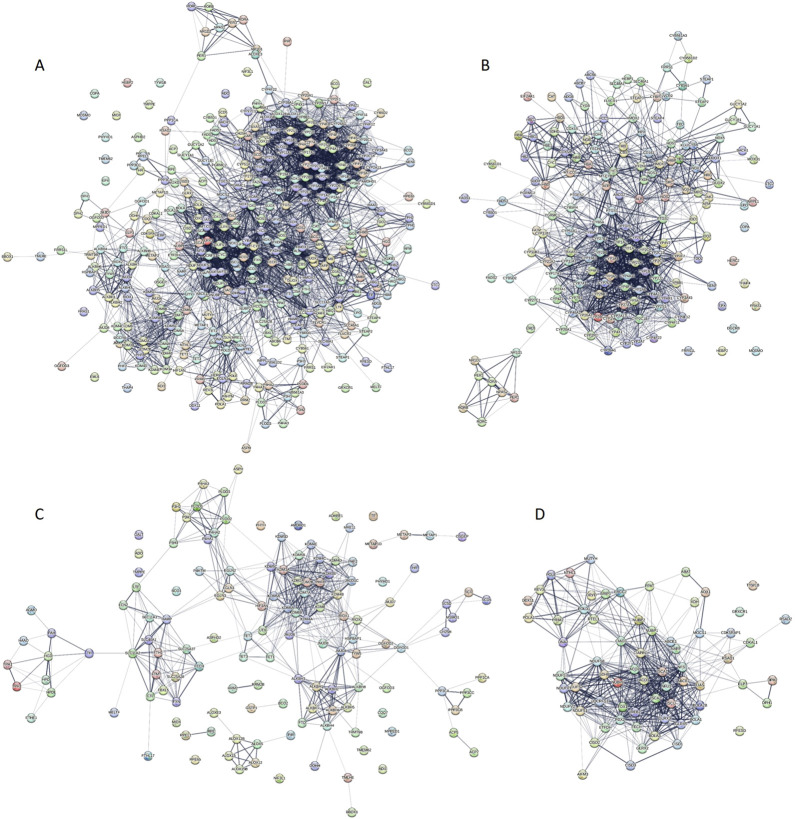
STRING-generated protein-protein interaction networks of entire human ironome **(A)** and sets of human heme- **(B)**, iron ion- **(C)** and iron-sulfur cluster-binding proteins **(D)**.

To explore the interconnectivity among human ironome members, we used the STRING platform to construct a protein-protein interaction (PPI) network. The network was generated using the default medium confidence threshold of 0.4 for the minimum interaction score. Edges in the network represent both functional and physical protein associations, with line thickness indicating the strength of supporting data (Figure 4A). Due to incomplete STRING data, not all human ironome members were represented. The resulting network comprised 372 proteins connected by 3,499 interactions. Among these, 18 proteins (ADO, ASPHD2, COPA, EML6, EPX, FRAS1, GALT, GRXCR1, HEBP2, MIOX, MOSMO, NIF3L1, PHYHD1, THAP4, TMEM62, TMPPE, and TYW1B) were isolated, showing no interactions with other ironome members. At the opposite end, 18 proteins each interacted with at least 50 other human ironome members including: FXN (50 partners), CYP2C9 (51 partners), FDX2 (51 partners), CYP4A11 (52 partners), CYB5A (54 partners), HMOX1 (56 partners), CYB5B (57 partners), ACO2 (58 partners), CYP2B6 (58 partners), ACO1 (59 partners), FECH (62 partners), CYP1A1 (63 partners), CYP2E1 (63 partners), CYP3A4 (63 partners), CYP1A2 (66 partners), CYCS (67 partners), ALB (74 partners), and FDX1 (87 partners). The network has an average node degree of 18.8 and an average local clustering coefficient of 0.508. Notably, this connectivity is significantly higher than expected for a random set of proteins of the same size and degree distribution, which would yield only 686 interactions (p-value <10^–16^).

To assess the robustness of our analysis, we varied the confidence score thresholds for STRING interactions. When the confidence of the minimum required interaction score was increased to 0.7 (high confidence), the number of edges decreased to 1168, the average node degree decreased to 6.28, and the average local clustering coefficient slightly increased (0.516). The number of isolated proteins (“loners”) also rose to 72 (see Supplementary Figure S1A). Further increasing the threshold to 0.9 (highest confidence) reduced the number of edges to 739, decreased the average node degree to 3.97, and slightly lowered the average local clustering coefficient to 0.484, while the number of loners increased to 127 (see Supplementary Figure S1B). We also examined the network under even stricter criteria—considering only physical interactions at the highest confidence level (0.9). In this scenario, the resulting PPI network consisted of 226 loners, with the remaining 146 proteins exhibiting node degrees between from 1 to 10 (see Supplementary Figure S1C). Among these, 20 proteins interacted with at least five partners: CYBA, CYBB, CYP2A6, CYP2J2, MT-CO1, NOX4, and NOX5 (each with five partners); MT-CYB, CYP1A2, DUOX1, NDUFS7, and NOX1 (each with 6), CYCS, NDUFS1, NDUFS8, NDUFV1, and NDUFV2 (each with 7); UQCRFS1 (8), and CYC1 (10). We then used these 146 interacting ironome members to generate a STRING network under the same stringent criteria (confidence level of 0.9, physical interactions only), where edges represent membership in a physical protein complex. This sub-network contained 176 edges, with an average node degree of 2.41 and a notably high average local clustering coefficient of 0.798 (see Supplementary Figure S1D).

Using the same default parameters—confidence level of 0.4, the full STRING network including both functional and physical protein associations, and edge line thickness indicating interaction confidence—we generated PPI networks for human heme-, iron ion- and iron-sulfur cluster-binding proteins. The resulting networks are presented in Figures 4B–D, respectively. The heme-binding protein network (Figure 4B) consists of 172 nodes with 1492 edges (see Figure 4B). While its average node degree (17.3) is slightly lower than that of the full ironome network, it has a higher average local clustering coefficient of 0.539. This network is significantly more interconnected than expected for a random set of proteins with similar size and degree distribution (expected number of edges = 157, p-value <10^–16^). The PPI network for iron ion-binding proteins (Figure 4C) includes 136 nodes and 525 edges, making it the least connected among the three. This is reflected in its lowest average node degree of 7.72 and a relatively high number of isolated proteins. Nonetheless, it maintains a high average local clustering coefficient of 0.615 and significantly exceeds the expected number of edges (59, p-value <10^–16^). As shown in Figure 4D, the human iron-sulfur cluster-binding protein network comprises 67 nodes and 551 edges. Despite its smaller size, it has a high average node degree of 16.4 and the highest local clustering coefficient among all networks analyzed (0.642). This network also shows a substantial enrichment in interactions compared to a random protein set of similar size (expected number of edges = 37, p-value <10^–16^).

Supplementary Tables S1 summarizes the major functional features of these networks showing their five most enriched biological processes, molecular functions, and cellular components (as per Gene Ontology annotations), as well as the most enriched local STRING network clusters, and KEGG pathways. These features were selected based on their lowest false discovery rates evaluated as p-values corrected for multiple testing within each category using the Benjamini–Hochberg procedure.

We also examined the functional enrichment of the most stringent network, composed of 146,146 ironome proteins involved in high-confidence level physical interactions (see Supplementary Figure S1D). The results, presented in Supplementary Figure S2, reveal that despite the reduced network size and high stringency, the functional enrichment profile closely mirrors that of the complete human ironome, as detailed in Supplementary Tables S1. While Supplementary Figure S2 highlights the top 10 enriched terms, the full set of statistically significant enrichments (false discovery rate <0.05, adjusted the Benjamini–Hochberg procedure) includes 301 Gene Ontology (GO) biological process terms, 129 GO molecular function terms, 37 GO cellular component terms, 45 local STRING network clusters, 52 KEGG pathways, and 52 disease-gene associations.

**FIGURE 5:**
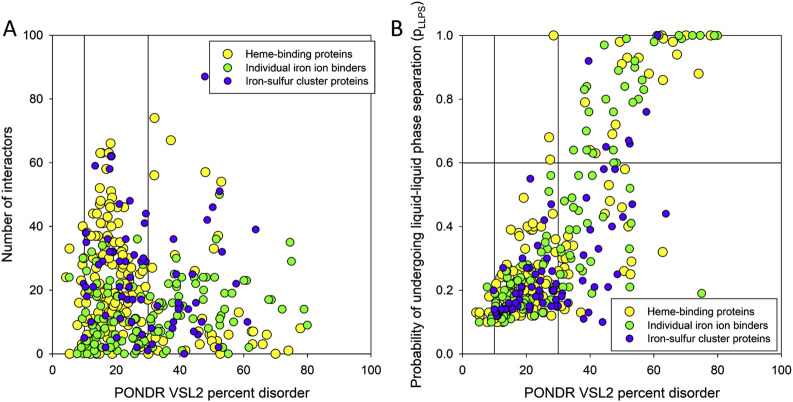
**(A)** Dependence of the number of interactors within the ironome PPI network on the PPIDR level of the human heme-, iron ion-, and iron-sulfur cluster-binding proteins. Vertical lines show 10% and 30% thresholds. **(B)** Comparison of the LLPS predisposition of the members of human ironome with their intrinsic disorder propensity. Vertical lines show 10% and 30% thresholds, whereas the horizontal line corresponds to the 0.6 p_LLPS_ threshold.

Interestingly, as illustrated in Figure 5A, although human heme-, iron ion-, and iron-sulfur cluster-binding proteins span a broad spectrum of intrinsic disorder and connectivity within the ironome PPI network, there is little correlation between their disorder levels and their degree of interactivity.

Liquid-liquid phase separation (LLPS) represents one of the specialized modes of protein interactions. In fact, under particular conditions, many proteins are capable of LLPS. Importantly, the LLPS predisposition of a given protein is correlated with its disorder status, with most proteins capable of spontaneous LLPS being characterized by the presence of high disorder levels (Uversky et al., 2015; Uversky, 2017a; b;Darling et al., 2019; Turoverov et al., 2019; Uversky and Finkelstein, 2019; Nesterov et al., 2021; Uversky, 2021; Fonin et al., 2022; Uversky, 2024). This is mostly because the IDPs/IDRs can be engaged in weak multivalent interactions, which represent an important feature required for proteins to undergo LLPS (Brocca et al., 2020; Uversky, 2021; Antifeeva et al., 2022). LLPS serves as a mechanistic foundation for the biogenesis of various membrane-less organelles (MLOs) or biomolecular condensates (Uversky, 2015; 2017a; b), which are very diverse and commonly found in cytoplasm, nucleus, mitochondria of various eukaryotic cells, in chloroplasts of plant cells, as well as in bacterial cells (Hyman et al., 2014; Toretsky and Wright, 2014; Alberti and Hyman, 2016; Chong and Forman-Kay, 2016; Courchaine et al., 2016; Saha et al., 2016; Alberti, 2017; Banani et al., 2017; Hnisz et al., 2017; Shin and Brangwynne, 2017; Uversky, 2017a; b;Forman-Kay et al., 2018; Zaslavsky and Uversky, 2018; Alberti and Dormann, 2019; Alberti et al., 2019; Gomes and Shorter, 2019). In fact, many proteins residing in MLOs and biomolecular condensates are intrinsically disordered, indicating that intrinsic disorder is important for the MLO biogenesis, and, more generally, for various emergent events taking place in a living cell (Uversky et al., 2015; Darling et al., 2019; Turoverov et al., 2019; Uversky and Finkelstein, 2019).

To check if the human ironome members can undergo spontaneous LLPS, we used the FuzDrop algorithm. The results of this analysis are summarized in Figure 5B as the p_LLPS_ vs. PPIDR plot, which shows that these proteins are characterized by highly diversified LLPS predispositions. In fact, 12.4%, 23.1%, and 8.8% of human heme-, iron ion-, and iron-sulfur cluster-binding proteins can serve as droplet-drivers (i.e., they are expected to be capable of spontaneous LLPS based on their FuzDrop-identified p_LLPS_ values exceeding the 0.6 threshold). On the other hand, 55.8%, 55.3%, and 67.7% of human heme-, iron ion-, and iron-sulfur cluster-binding proteins can act as droplet-clients, being characterized by the p_LLPS_ values below 0.6, but containing droplet-promoting regions (DPRs), which are defined as regions that contain at least five consecutive residues with the residue-based droplet-promoting probabilities p_DP_ ≥ 0.60. The remaining 31.8%, 21.6%, and 23.5% of the corresponding sets are not related to LLPS, as they have low p_LLPS_ values and do not contain DPRs. These numbers of droplet-drivers in various sets of iron-binding proteins are noticeably lower that that reported for the entire human proteome, where about 40% or proteins were predicted as “droplet-driving” proteins expected to undergo spontaneous LLPS under physiological conditions (Hardenberg et al., 2020). However, this is a rather expected outcome taking into account that ironome is noticeably more ordered than entire proteome. Curiously, Figure 5B shows that not all highly disordered members of the human ironome serve as droplet drivers, with 47.5% of highly disordered heme- and iron ion-binding proteins not acting as droplet drivers, and with this value increasing to 70% for highly disordered human iron-sulfur cluster-binding proteins.

Surprisingly, despite the wealth of information on various aspects pertaining to the LLPS and MLOs/biomolecular condensates, current literature discussing phase separation of iron-binding proteins is sparse. It was reported that strongly basic bovine lactoferrin is able to form coacervates (biomolecular condensates) with a weakly acidic β-lactoglobulin (Yan et al., 2013; Kizilay et al., 2014; Flanagan et al., 2015; Peixoto et al., 2016). Another interesting case is given by the phase separation of ferritin driven by the nuclear receptor coactivator 4 (NCOA4) (Ohshima et al., 2022). Ferritin is an important iron storage protein, which forms particles or ferritin cores containing 24 ferritin heavy chain 1 (FTH1) and ferritin light chain (FTL) subunits, and which incorporates up to 4,500 Fe^2+^ ions, oxidizes them to Fe^3+^, and stores them as mineral cores (Mann et al., 1986). Formation of ferritin condensates was shown to depend on multivalent interactions between the ferritin particles and NCOA4 homodimers (Ohshima et al., 2022; Gehrer et al., 2023). Recently, it was also shown that transferrin, which is the major iron-binding protein in the blood that regulates iron absorption to the blood and transports it throughout the body, is capable of LLPS, and the phase separated condensates of this protein completely prevented its pathological aggregation and fibrillation (Patel et al., 2023). An interesting example is given by tau protein that has an iron-binding motif and is involved in iron transport within neurons (Rao and Adlard, 2018). Furthermore, it was shown that ferric (Fe^3+^) ions promoted tau LLPS and enhanced its aggregation and fibrillation, whereas ferrous (Fe^2+^) ions did not affect tau droplet formation nor increased its aggregation (Mukherjee and Panda, 2021). These observations are in line with earlier studies showing that Fe^3+^ is associated with neurofibrillary tangles (NFTs) in Alzheimer’s disease (AD) and progressive supranuclear palsy (PSP) and promotes aggregation of hyperphosphorylated tau, whereas the aggregation of tau can be reversed and tau species isolated from AD brains can be solubilized by reducing Fe^3+^ to Fe^2+^ (Smith et al., 1997; Yamamoto et al., 2002; Rao and Adlard, 2018). Curiously, another iron-binding protein, ferritin, was shown to interact with the aberrant tau filaments present in PSP (Perez et al., 1998). Another famous IDP associated with the pathogenesis of Parkinson’s disease (PD), α-synuclein, was also shown to be involved in iron metabolism, e.g., acting as a cellular ferrireductase (Davies et al., 2011; McDowall et al., 2017; Sian-Hulsmann and Riederer, 2020). Similar to tau, Fe^3+^ was shown to accelerate the LLPS of α-synuclein (Ray et al., 2020; Sawner et al., 2021).

Although the focus of this study is on the ironome, some complementary insights can be gained from the consideration of the information related to the established capability of calcium-binding proteins to act as LLPS regulators. Some illustrative examples of the interplay between LLPS and calcium ions are given below. It was indicated that the Ca^2+^-dependent LLPS of specific calcium-binding proteins may contribute to the action of cellular Ca^2+^ stores, such as endoplasmic reticulum (ER) and sarcoplasmic reticulum (SR), providing an intricate regulation mechanism of Ca^2+^ storage and release within the ER/SR (Mayfield et al., 2021). This hypothesis is based on the important observation that the Ca^2+^-sequestering protein, calsequestrin-1 (CASQ1) is capable of forming biomolecular condensates in a Ca^2+^-dependent manner, with the process being further regulated *via* CASQ1 phosphorylation by the secretory pathway kinase FAM20C (Mayfield et al., 2021). On a similar note, temporary fluctuations or oscillations in the concentration of intracellular calcium ions (so-called Ca^2+^ transients) on the outer surface of the ER membrane were shown to stimulate LLPS of FIP200 (FAK family kinase-interacting protein of 200 kDa also known as RB1-inducible coiled-coil protein 1) leading to the formation of liquid-like FIP200 puncta for assembly of the autophagosome initiation complex on the ER in mammalian cells (Zheng et al., 2022). A calcium sensor protein, synaptotagmin-1 (Syt1), which is involved in regulation of the synaptic vesicle fusion in synchronous neurotransmitter release, was shown to undergo LLPS to form Syt1 condensates participating in vesicle attachment to the plasma membrane and recruitment of SNAREs (SNAP (synaptosomal-associated protein) REceptors) and complexin (Zhu et al., 2024). Curiously, these Syt1 condensates were shown to undergo a liquid-to-gel-like phase transition which was efficiently blocked or reversed by Ca^2+^ (Zhu et al., 2024). LLPS of Ca^2+^-binding IDPs plays a crucial role in calcium carbonate biomineralization (Tarczewska et al., 2022). For example, the intrinsically disordered aspartic and glutamic acid-rich protein (AGARP) from the Great Barrier Reef scleractinian coral *Acropora millepora* was shown to significantly affect early stages of CaCO_3_ nucleation and crystal growth through LLPS (Klepka et al., 2024). Size and shape of the nuage/chromatoid body condensates changed after exposure to Ca^2+^ during spermatogenesis (Andonov and Chaldakov, 1989). Ca^2+^ binding was shown to regulate stability, internal mobility, and interfaces of DEAD box helicase condensates, and also affect selective partitioning of molecules into those condensates (Crabtree et al., 2023). All these examples clearly show that Ca^2+^ can act as an efficient regulator of phase separation of calcium-binding proteins.

Concluding this part, our analysis revealed that although human ironome is significantly less disordered than the human calceome many ironome members contain appreciable levels of intrinsic disorder. To get an idea on what roles intrinsic disorder may play in human iron-containing proteins, we conducted an in-depth analysis of the most disordered representatives of human heme- (UniProt IDs: O15534 and O14867), iron ion- (UniProt IDs: O43151 and O15054), and iron-sulfur cluster-binding proteins (UniProt IDs: Q6FI81 and O60673). Results of these analyses are summarized below.

### 3.2 Illustrative examples of the functional importance of intrinsic disorder in the most disordered members of the human ironome

IDPs/IDRs are recognized as multifunctional promiscuous binders, which are commonly involved in recognition and signaling, as well as regulation and control of various cellular processes (Uversky et al., 2000; Dunker et al., 2001; Dunker et al., 2002a; Dunker et al., 2002b; Uversky, 2013a; Habchi et al., 2014; van der Lee et al., 2014). Structural “floppiness” provides multiple functional advantages of IDPs/IDRs over the ordered proteins, and also forms a foundation for the multitude of multi-level mechanisms of their functional regulation and control (Uversky, 2011; 2013b; Habchi et al., 2014; van der Lee et al., 2014), e.g., by utilization of various post-translational modifications (PTMs) (Iakoucheva et al., 2004; Pejaver et al., 2014; Darling and Uversky, 2018), alternative splicing (Romero et al., 2006), interaction with numerous binding partners of different physico-chemical nature and mutations. Some IDPs/IDRs can bind to multiple partners *via* gaining very different structures in the bound state, and this adjustable promiscuity represents an important means for the increased complexity of the disorder-based interactomes (Oldfield et al., 2008; Alterovitz et al., 2020).

#### 3.2.1 Highly disordered heme-binding proteins

##### 3.2.1.1 Period circadian protein homolog 1 (UniProt IDs: O15534, PPIDR_PONDR VSL2_ = 77.83%)

Human period circadian protein homolog 1 (hPER1, also known as RIGUI) is a 1290-residue-long heme-binding transcriptional repressor, which plays a crucial role in the formation of a core component of the circadian clock, by helping maintaining the cellular circadian rhythms, and, if dysfunctional, by likely playing a role in cancer development (Gery et al., 2006; Lamont et al., 2007; Yang et al., 2009). Although hPER1 is expressed in the suprachiasmatic nucleus (SCN) that acts as the primary circadian pacemaker in the brain, it is also expressed throughout mammalian peripheral tissues (Lamont et al., 2007) including the hair follicle, where it is involved in the regulation and modulation of the human hair cycle clock (Al-Nuaimi et al., 2014). hPER1 is a circadian oscillator, and the transcription of its gene is also rhythmic with a period of approximately 24 h (Sun et al., 1997). In relation to the subject of our study, it was shown that cellular iron depletion enhances behavioral rhythm by limiting brain *Per1* expression in mice (Wu et al., 2024). Furthermore, the expression of genes involved in iron deficiency response and iron storage, such as *IRT1*, *bHLH39*, and *FER1*, is regulated by the circadian clock (Hong et al., 2013). In addition, some circadian clock proteins, including those related to hPER1 (e.g., hPER2), were shown to possess domains (e.g., PAS domain of hPER2) capable of heme binding, suggesting that they may act as sensors for the changes in oxygen levels that could affect circadian rhythms (Kaasik and Lee, 2004; Kitanishi et al., 2008; Yang et al., 2008).

**FIGURE 6:**
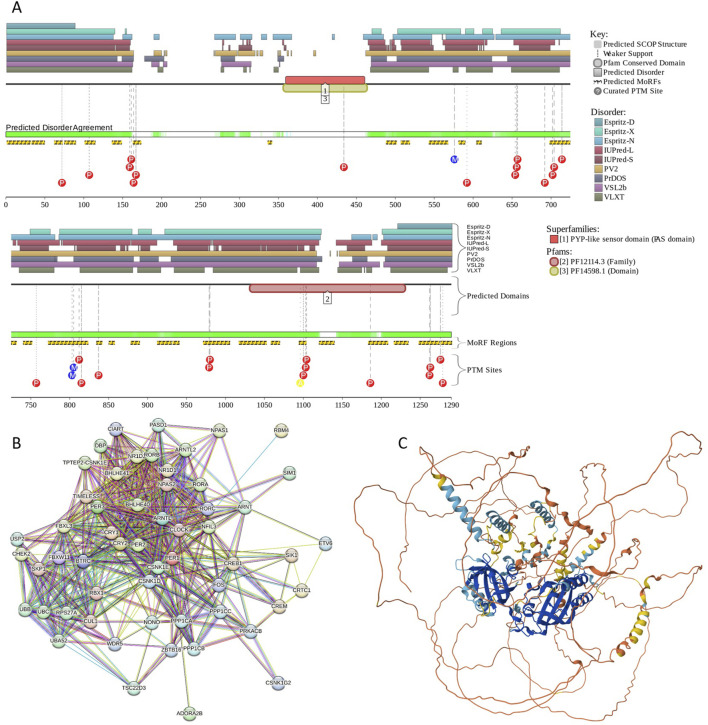
Functional disorder analysis of human PER1 (UniProt IDs: O15534). **(A)** Functional disorder profile generated by the D^2^P^2^ platform. Here, the IDR localization predicted by IUPred, PONDR® VLXT, PONDR® VSL2, PrDOS, PV2, and ESpritz is shown with nine differently colored bars at the top of the plot, whereas the agreement between the outputs of these disorder predictors is indicated by the middle green-white bar, with the consensus disordered regions shown in blue and green. The two lines with colored and numbered bars above the disorder consensus bar show the positions of functional SCOP domains (Murzin et al., 1995; Andreeva et al., 2004) predicted using the SUPERFAMILY predictor (de Lima Morais et al., 2011). Positions of the predicted disorder-based binding sites (MoRF regions) identified by the ANCHOR algorithm are shown by yellow zigzagged bars (Meszaros et al., 2009). Locations of the sites of different posttranslational modifications (PTMs) identified by the PhosphoSitePlus platform (Hornbeck et al., 2012) are shown at the bottom of the plot with the differently colored circles. **(B)** Protein-protein interaction (PPI) network of human PER1 generated by STRING using seven types of evidence shown by differently colored lines: a black line represents co-expression evidence; a blue line–co-occurrence evidence; a green line - neighborhood evidence; a light blue line–database evidence; a purple line–experimental evidence; a red line–the presence of fusion evidence; and a yellow line–text mining evidence (Szklarczyk et al., 2011). **(C)** 3D structure for PER1 as modeled by AlphaFold. Structure is colored based on the AlphaFold-generated per-residue confidence score (p_LDDT_) that ranges between 0 and 100, where orange, yellow, cyan, and blue colors correspond to the segments predicted by AlphaFold with very high very low (p_LDDT_ < 50), low (70 > p_LDDT_ > 50), high (90 > p_LDDT_ > 70), and (p_LDDT_ > 90) confidence.

Human hPER1 protein has a complex domain structure, which is not surprising for a protein of its size. It encompasses a region involved in interaction with F-box/WD repeat-containing protein 1A (also known as BTRC, residues 1–151) (Shirogane et al., 2005), nuclear export signals (residues 138–147, 489–498, and 982–989), a nuclear localization signal (residues 827–843), two PAS domains (residues 208–278 and 348–414), a PAC domain (residues 422–465), regions required for phosphorylation by CSNK1E (residues 596–815), a LXXLL motif (residues 1043–1047), and a CRY-binding domain (residues 1207–1290). Figure 6A shows that most of the hPER1 protein is predicted as intrinsically disordered. This high level of intrinsic disorder explains the lack of structural information for this important protein. Figure 6A also illustrates that hPER1 possesses multiple disorder-based binding sites, known as molecular recognition features (MoRFs), which are disordered regions that undergo a disorder-to-order transition at interaction with specific binding partners. In fact, MoRFs represent one of the molecular mechanisms of the utilization of intrinsic disorder in protein interactability (Katuwawala et al., 2019). The BTRC-binding domain includes 7 MoRFs (residues 1-31, 33-49, 62-72, 75-89, 101–114, 136–148, and 163–173). Although both PAS domains are predicted as mostly ordered, the linker between these domains includes another MoRF (residues 336–341). The C-terminal two-thirds of PER1 are mostly disordered and highly enriched in MoRFs and sites of various posttranslational modifications, which are commonly located within IDRs (Darling and Uversky, 2018). Further, while the region required for phosphorylation by CSNK1E covers 5 MoRFs (residues 581–589, 604–610, 698–731, 740–751, and 772–823), the entire CRY-binding domain represents a long, disordered, binding platform with 4 MoRFs spanning almost its entire length (residues 1183–1208, 1216–1234, 1248–1274, and 1276–1290). With so many MoRFs, one can expect PER1 to acts as a highly promiscuous binder. This hypothesis is supported by the results in Figure 6B, which illustrate the PPI network centered at hPER1. This network includes 56 nodes linked *via* 519 edges and is characterized by the average node degree of 18.5 and average local clustering coefficient of 0.738. The 3D structure of hPER1 modeled by AlphaFold is shown in Figure 6C and supports the notion that a very significant portion of this protein does not have stable structure.

Since intrinsically disordered proteins are commonly involved in liquid-liquid phase separation that serves as a mechanistic foundation of the biogenesis of various membrane-less organelles, we evaluated the LLPS predisposition hPER1 using the computational platform FuzDrop (Hardenberg et al., 2020; Hatos et al., 2022; Vendruscolo and Fuxreiter, 2022). This analysis revealed that hPER1 is characterized by p_LLPS_ = 1.00 and contains 12 DPRs (residues 1–143, 268–287, 344–363, 463–482, 492–546, 567–607, 640–707, 744–782, 794–980, 990–1121, 1161–1182, and 1195–1290. Although there is no currently available information on the experimentally validated LLPS of the hPER1-containing solutions, in *Neurospora*, the intrinsically disordered RNA-binding protein PERIOD-2 was shown to form ordered gel-like assemblies *in vitro* and micrometer-scale assemblies *in vivo* that behave as liquid droplets, being deformable and capable to fuse (Bartholomai et al., 2022). It was suggested that PERIOD-2 mediates clock-regulated perinuclear localization of clock gene RNAs within the circadian cycle of *Neurospora* (Bartholomai et al., 2022). It is tempting to hypothesize that hPER1 might also have LLPS-dependent functionality.

It was established that due to the alternative splicing at least three different transcripts can originate from human *PER1/RIGUI* gene (Sun et al., 1997). These transcripts were described as *RIGUI 3.0*, *RIGUI 4.7,* and *RIGUI 6.6*, and their encoded protein diverged at the C-terminal portions. *RIGUI 4.7* encodes the canonical form of hPER1, whereas *RIGUI 6.6* gave a protein of 875 residues, whose initial 821 residues were identical to those of hPER1, but the remaining residues diverged, and *RIGUI 6.6* encoded for a 798-residue-long polypeptide, which diverged from hPER1 at residue 758 (Sun et al., 1997). Unfortunately, functionalities of the alternatively splicing variants have not been fully described.

##### 3.2.1.2 Transcription regulator protein BACH1 (UniProt IDs: O14867, PPIDR_PONDR VSL2_ = 74.05%)

BTB and CNC homolog one or transcription regulator protein BACH1 is a 736 residue-long heme-dependent transcriptional regulator (Blouin et al., 1998) that acts as a repressor or activator, depending on the context, being, e.g., involved in repression of the transcription of genes under the control of the NFE2L2 oxidative stress pathway (Tan et al., 2013). By forming heterodimers with the small Maf proteins, such as MafK (Oyake et al., 1996), BACH1 acts as a repressor of the enhancers of heme oxygenase-1 (HO-1) gene (*Hmox-1*) (Sun et al., 2002; Hira et al., 2007). The heme-mediated regulation of BACH1 is controlled by cysteine-proline dipeptide motifs (CP motifs) (Hira et al., 2007), with six such motifs being present in human BACH1 (residues 224–225, 299–300, 435–436, 461–462, 492–493, and 646–647). BACH1 belongs to the Cap “n” Collar and basic region Leucine Zipper (CNC-bZIP) family, which is broadly expressed in various mammalian tissues and is involved in epigenetic modifications, heme homeostasis, oxidative stress, and immune system development (Hu et al., 2024). Furthermore, it plays crucial roles in inflammatory diseases and cancer (Hu et al., 2024).

**FIGURE 7:**
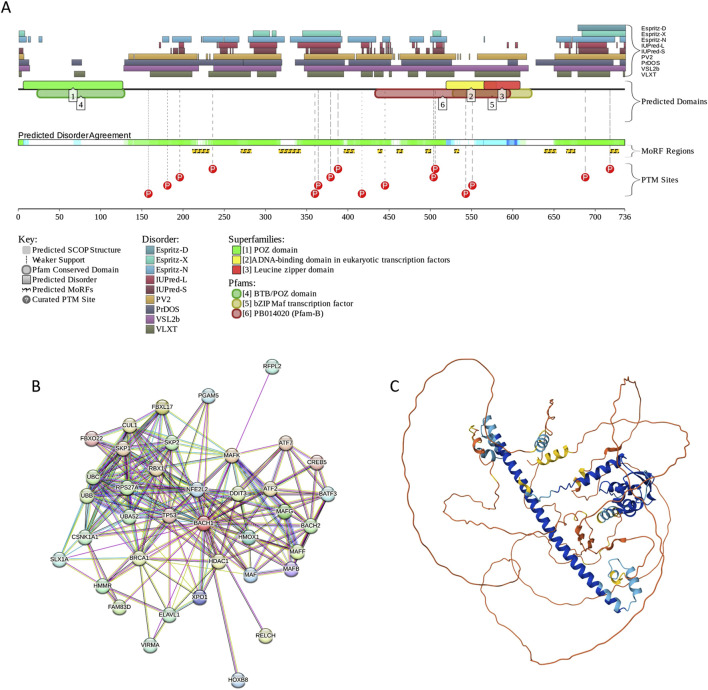
Functional disorder analysis of human BACH1 (UniProt IDs: O14867). **(A)** Functional disorder profile produced by the D^2^P^2^ platform. **(B)** STRING-generated PPI network centered at BACH1. **(C)** 3D structure for BACH1 as modeled by AlphaFold. Structure is colored based on the pLDDT values.

BACH1 possesses several functional regions, such as Broad-complex, Tramtrack, and Bric-à-brac (BTB) domain (residues 34–100), six CP motifs, a bZIP domain (residues 557–620) containing a leucine zipper (residues 582–589), and a cytoplasmic localization signal (CLS, residues 685–725). The protein is predicted to have high disorder content, multiple MoRFs (residues 211–231, 270–282, 316–342, 395–407, 436–441, 459–466, 494–500, 529–534, 638–652, 665–675, and 718–728), and numerous PTM sites (see Figure 7A). Although the BTB domain, which is involved in the regulation of gene transcription by interacting with non-BTB proteins and binding to the chromatin structure (Albagli et al., 1995), is predicted to be mostly ordered, noticeable levels of disorder are found in the DNA-binding bZIP domain (see Figure 7A). High content of MoRFs predisposes BACH1 for disorder-based interactivity, which is reflected in the BACH1-centered PPI network generated by STRING, where 36 proteins are involved in 199 interactions, forming a dense cluster with the average local clustering coefficient of 0.73 (see Figure 7B). The average node degree of this network is 10.5, and it includes significantly more interactions (i.e., 199) than expected for a random set of proteins of the same size and degree distribution drawn from the genome (p-value = 1.11 × 10^−16^), which is 104 interactions. BACH1 is known to establish binary interactions with 11 proteins, such as CREB1 (*via* residues 267–327), ATF1, ATF2, ATF3, BATF, and FOS (*via* residues 574–634), and MAF, MAFB, MAFF, MAFG, MAFK, and DDIT3 (*via* residues 548–624), as well as forming a homodimer *via* the residues 7–128 that includes the BTB domain. Although the crystal structure of the dimeric form of the BTB domain was solved (PDB ID: 2IHC), the remaining part of protein is predicted to be highly disordered, as evidenced by Figure 7A and further supported by the AlphaFold-generated structural model (Figure 7C).

According to the FuzDrop-based analysis, BACH1 is predicted to have p_LLPS_ of 0.8828 and contain 8 DPRs, residues 188–215, 233–244, 275–321, 346–390, 421–432, 501–518, 653–667, and 684–721. These observations indicate that BACH1 can act as a droplet-driver, being potentially able to spontaneously undergo liquid-liquid phase separation. Unfortunately, current literature does not contain information on the LLPS behavior of BACH1.

It was shown that the alternative splicing of human *BACH1* gene produces the *BACH1t* isoform encoding a truncated form of BACH1 protein that lacked the leucine zipper domain, but retained the BTB domain, CNC domain, and basic region (Kanezaki et al., 2001). Noticeable functional diversification was reported to be caused by alternative splicing. In fact, despite similar expression of both *BACH1* and *BACH1t* transcripts in all tissues, the encoded proteins possessed different cellular localization, with the full-length BACH1 being exclusively found in the cytoplasm, and with the BACH1t being accumulated in the nucleus (Kanezaki et al., 2001). However, efficient nuclear accumulation of BACH1 was induced *via* the BTB/POZ domain-driven formation of the BACH1t-BACH1 hetero-oligomers, indicating that BACH1t recruits BACH1 to the nucleus (Kanezaki et al., 2001).

#### 3.2.2 Highly disordered iron ion-binding proteins

##### 3.2.2.1 Methylcytosine dioxygenase TET3 (UniProt IDs: O43151, PPIDR_PONDR VSL2_ = 80.00%)

Methylcytosine dioxygenase TET3 is a 1795-residue-long member of the ten-eleven translocation (TET) family, whose members are Fe^2+^-dependent dioxygenases that use α-ketoglutarate (α-KG) to catalyze DNA demethylation. In human TET3, catalytic Fe^2+^ is coordinated by residues 1077, 1079, and 1673. In addition, several Zn^2+^ ions are used as co-factors, however, mostly with a structural role. These Zn^2+^ ions are coordinated by residues 57, 60, 63, 89, and 914 (Zn^2+^ 1), 69, 72, 75, 84 and 1,075 (Zn^2+^ 2), 828, 830, 916, 984, 993, 1054, and 1704 (Zn^2+^ 3), and 988, 966, and 968.

TET3 plays a number of key roles in the epigenetic chromatin reprogramming in the zygote following fertilization being responsible for catalysis of the conversion of the modified genomic base 5-methylcytosine (5 mC) into the 5-hydroxymethylcytosine (5hmC), 5-formylytosine (5 fC), and 5-carboxylcytosine (5caC) to achieve DNA demethylation (Beck et al., 2020). It also can contribute to the regulation of the expression of numerous developmental genes by selectively binding to the promoter region of target genes (Xu et al., 2012). Furthermore, TET3 promotes histone H2B GlcNAcylation by OGT by being involved in the O-GlcNAc transferase OGT recruitment to the CpG-rich transcription start sites of active genes (Deplus et al., 2013). It was also pointed out that TET proteins participate in the oxidation of 5-methylcytosine in RNA (Fu et al., 2014; Delatte et al., 2016; Basanta-Sanchez et al., 2017). Similar to other members of the TET family, TET3 is related to several conserved signaling pathways in various organs and tissues during development, especially in embryo and cancer development (Mahajan et al., 2020; Woods et al., 2020). For example, it was emphasized that the members of the family of TET enzymes may play crucial roles in the immune microenvironment at the maternal-fetal interface during decidualization and early pregnancy (Jin et al., 2022).

**FIGURE 8:**
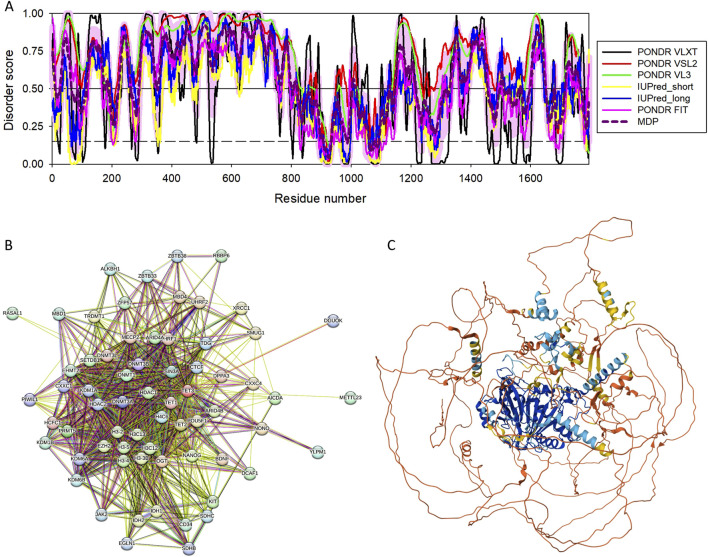
Functional disorder analysis of human TET3 (UniProt IDs: O43151). **(A)** Per-residue disorder profile generated by RIDAO. The outputs of PONDR® VLXT, PONDR® VSL2, PONDR® VL3, PONDR® FIT, IUPred long, and IUPred short are shown by black, red, green, pink, blue, and yellow lines, respectively. Mean disorder profile (or mean disorder prediction, MDP) calculated as an average of outputs of these six predictors is shown by dashed dark pink line, whereas error distribution are shown as light pink shadow. In this per-residue disorder analysis, a disorder score was assigned to each residue. A residue with disorder score equal to or above 0.5 is considered as disordered and a residue with disorder score below 0.5 is predicted as ordered. Residues/regions with disorder scores between 0.15 and 0.5 were considered as ordered but flexible. The corresponding thresholds are shown by solid (0.5) and long-dashed lines (0.15). **(B)** Protein-protein interaction (PPI) network of human TET3 generated by STRING using seven types of evidence shown by differently colored lines: a black line represents co-expression evidence; a blue line–co-occurrence evidence; a green line - neighborhood evidence; a light blue line–database evidence; a purple line–experimental evidence; a red line–the presence of fusion evidence; and a yellow line–text mining evidence (Szklarczyk et al., 2011). **(C)** 3D structure for TET3 as modeled by AlphaFold. Structure is colored based on the p_LDDT_ values.

The human TET3 protein contains several functional regions, such as the CXXC domain (residues 46–102), and a catalytic C-terminal region that includes a cysteine-rich insert (residues 825–1011), a DSBH domain that is comprised of a double-stranded β helix, also known as the jelly-roll motif (residues 1012–1719) with the low complexity insert (residues 1159–1635) (Akahori et al., 2015). Despite its functional and pathological importance, human TET3 remains mostly structurally uncharacterized. In fact, the 3D structure is currently known only for the short zinc-finger region of TET3 (residues 49-98) containing the CXXC domain bound to the CpG DNA [PDB ID: 4Z3C; (Xu et al., 2018)]. This is because this protein is predicted to be highly disordered. Since D2P2 does not contain TET3-related information, we evaluated the intrinsic disorder predisposition of this protein using RIDAO, which combines the outputs of six commonly used per residue disorder predictors, such as PONDR® FIT, PONDR® VSL2, PONDR® VL3, PONDR® VLXT, IUPred Short, and IUPred Long to generate an integral disorder profile of an individual query protein or to provide global disorder characterization of a protein dataset (Dayhoff and Uversky, 2022). Figure 8A represents the RIDAO-generated disorder profile of human TET3 and shows that a very significant part of this protein is expected to be disordered. To gain an information on the presence of disorder-based binding sites in this protein, we used UniProt2A platform (Meszaros et al., 2018; Erdos and Dosztanyi, 2020) that provides a possibility to find such regions utilizing the ANCHOR algorithm (Meszaros et al., 2009). This analysis revealed that TET3 has 15 MoRFs (residues 117–228, 250–266, 280–371, 382–574, 577–777, 790–808, 1140–1148, 1320–1444, 1450–1477, 1479–1496, 1524–1547, 1548–1560, 1574–1617, 1628–1664, and 1705–1708). Interactability of human TET3 is illustrated by STRING-generated PPI network, where one can find 66 node connected by 867 nodes (see Figure 8B). The average node degree of this network is 26, and it is characterized by the average local clustering coefficient of 0.765. The network includes significantly more interactions than the expected 207 interactions for a random set of proteins of the same size and degree distribution drawn from the genome (p-value <10^–16^). In line with the RIDAO-generated disorder profile, Figure 8C represent the modeled 3D structure of this protein and shows that it includes a very significant portion of unstructured regions.

Evaluation of the LLPS potential of human TET3 with FuzDrop revealed that this protein has a very high probability of spontaneous LLPS, p_LLPS_ = 0.9998 and possesses 15 DPRs (residues 28-55, 102–190, 213–254, 294–325, 362–461, 479–521, 531–706, 716–753, 772–792, 802–822, 1196–1220, 1248–1258, 1278–1513, 1560–1572, and 1593–1634. Although these data indicated that TET3 can spontaneously undergo LLPS, related information is not present in the current.

Due to the alternative use of promoters, which are regulated by the alternative activation of enhancers, and alternative splicing, human *TET3* gene encodes three isoforms (Joshi et al., 2022). One of the two shorter isoforms of TET3 (neuronal TET3) was shown to lack CXXC domain and therefore for its DNA binding, it is primarily dependent on interaction with other DNA-binding proteins (Jin et al., 2016; Joshi et al., 2022). For example, it was established that the transcriptional regulator REST specifically interacts with the neuronal TET3 isoform and elevates its hydroxylase activity (Perera et al., 2015). Furthermore, UniProt reports two other alternatively spliced TET3 isoforms, which differ from the canonical 1795-residue-long protein (isoform 1) by missing regions 1575–1690 (isoform 2) and 863–1795 (isoform 3). These sequence alterations result in the elimination of the portion of a low complexity insert and a C-terminal portion of the DSBH domain in the isoform 2, and almost completely remove a catalytic C-terminal region in isoform 3.

##### 3.2.2.2 Lysine-specific demethylase 6B (UniProt IDs: O15054, PPIDR_PONDR VSL2_ = 74.56%)

Lysine-specific demethylase 6B (KDM6B, also known as Jumonji domain-containing protein 3 (JMJD3) or [histone H3]-trimethyl-L-lysine(27) demethylase 6B) is a 1643-residue-long histone demethylase with a central role in the histone code *via* its ability to specifically demethylate trimethylated and dimethylated Lys-27 of histone H3 (Wirth, 1995; De Santa et al., 2007; Hong et al., 2007; Lan et al., 2007). It also regulates *HOX* expression, thereby playing a crucial role in regulation of posterior development (Lan et al., 2007), and, in the case of inflammation, KDM6B contributes to the inflammatory response by regulating gene expression and macrophage differentiation (De Santa et al., 2007). In addition to acting as a demethylase, this protein was shown to catalyze sequential oxidations accepting multiple N^ε^-alkylated lysine analogues and forming alcohol, aldehyde and carboxylic acid products (Hopkinson et al., 2018). Furthermore, KDM6B can also interact with other proteins than histones [e.g., it can interact with p53 and is recruited to p53 bound promoters and enhancer elements in a p53 dependent manner (Williams et al., 2014)].

**FIGURE 9:**
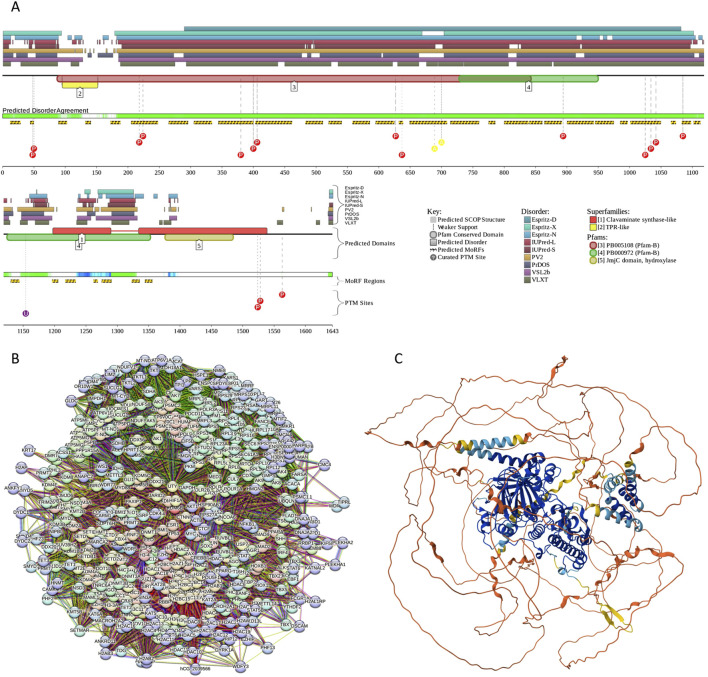
Functional disorder analysis of human KDM6B (UniProt IDs: O15054). **(A)** Functional disorder profile produced by the D^2^P^2^ platform. **(B)** STRING-generated PPI network centered at KDM6B. **(C)** 3D structure modeled by AlphaFold.

The catalytic activity of this histone demethylase depends on the two-oxoglutarate (2OG), ferrous iron, and oxygen. An important catalytic role is played by Fe^2+^ coordinated by residues 1390, 1392, and 1470 of the catalytic JmjC domain (residues 1339–1502), which is a conserved domain found from bacteria to human. Although several crystal structures were solved for the C-terminally located catalytic domain of KDM6B (residues 1141–1643) alone or in complex with various substrate peptides (e.g., PDB IDs: 6F6D and 5OY3 (Jones et al., 2018), 5FP3 (Westaway et al., 2016), 4ASK and 2XUE (Kruidenier et al., 2012), and 2XXZ), remaining 1140 residues remain structurally uncharacterized. Figure 9A provides an explanation for this observation showing that first 1100 residues of this protein are mostly disordered. D^2^P^2^ also clearly shows that this mostly disordered region contains multiple PTM sites and holds 28 MoRFs covering almost its entire length. Furthermore, several MoRFs are also located within the catalytic C-terminal region. Therefore, these observations suggest that KDM6B can act as a highly promiscuous binder, a notion strongly supported by Figure 9B representing an enormous PPI network centered at this protein. In fact, this dense network with the average clustering coefficient of 0.682 contains 472 proteins connected by 22,687 interactions. The average node degree of this PPI network reaches the colossal value of 96.1, indicating that, on average, members of this network interact with 96 partners each. The 22,687 interactions found in this network significantly exceed the 9,375 interactions expected for a random set of proteins of the same size and degree distribution drawn from the genome (p-value <10^–16^). Figure 9C represents the AlphaFold-modeled 3D structure for the human KDM6B protein and shows that a loosely structured (or unstructured) segment contributes to a very significant part of the protein, thereby providing further support to the disorder profile reported in Figure 9A.

FuzDrop-based analysis showed that KDM6B has 10 DPRs (residues 33-92, 184–497, 504–682, 701–803, 818–912, 916–952, 977–1112, 1175–1186, 1282–13.24, and 1382–1393). With the p_LLPS_ of 0.9999, human KDM6B is definitely capable of spontaneous LLPS. In line with these observations, a recent study revealed that KDM6B/JMJD3 is involved in the formation of phase-separated biomolecular condensates, which are found in the enhancer-cluster (i.e., multiple enhancers arrange in the 3D-space to control the activation of a specific promoter) (Vicioso-Mantis et al., 2022). It was also shown that KDM6B/JMJD3 can undergo LLPS both *in vitro* and *in vivo*, and that the condensate formation is dependent on the IDR of this protein (Vicioso-Mantis et al., 2022). This study described “an unforeseen role of TGFβ reorganizing the chromatin fiber in a JMJD3 histone demethylase-dependent manner. JMJD3 promotes the establishment of enhancer–enhancer and enhancer–promoter contacts that ultimately modulate *Chst8* enhancer activity, and thus the NSCs [neural stem cell] gene expression program” (Vicioso-Mantis et al., 2022).

Human KDM6B exists as two alternatively spliced isoforms, with longer isoform one being different from the canonical 1643-residue-long form by the presence of the C-terminal insertion following residue Leu1636 comprising residues VRARRARGQRRRALGQAAGTGFGSPAAPFPEPPPAFSPQ (Lagunas-Rangel, 2021) and originating from retaining of an intron in the 3′coding region of the corresponding mRNA. This insertion is predicted to be completely disordered and adds a new DPR (residues 1641–1682) and a new MoRF (residues 1655–1682), thereby affecting functionality of this important protein.

#### 3.2.3 Highly disordered iron-sulfur cluster-binding proteins

##### 3.2.3.1 Anamorsin (UniProt IDs: Q6FI81, PPIDR_PONDR VSL2_ = 63.78%)

Anamorsin, also known as cytokine-induced apoptosis inhibitor 1 (CIAPIN1) and Fe-S cluster assembly protein DRE2 homolog, is a 312-residue-long protein acting as an important component of the cytosolic iron-sulfur (Fe-S) cluster protein assembly (CIA) machinery, which is crucial for the maturation of extramitochondrial Fe-S cluster-containing proteins (Banci et al., 2013). Besides its well-documented, crucial roles in the assembly of the [4Fe-4S] clusters and the assembly of the diferric tyrosyl radical cofactor of ribonucleotide reductase (RNR), anamorsin/CIAPIN1 has anti-apoptotic effects, promotes development of hematopoietic cells, and upon cytokine withdrawal, is involved in the negative control of cell death (Banci et al., 2013).

**FIGURE 10:**
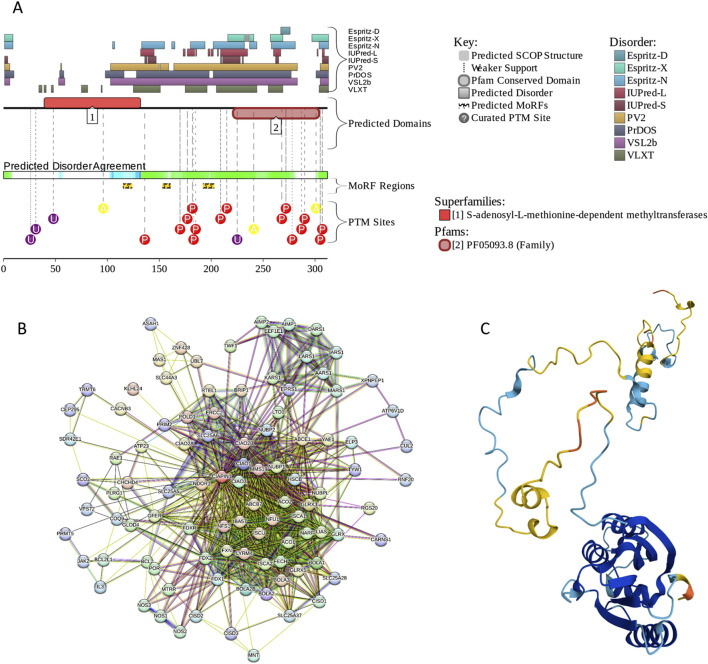
Functional disorder analysis of human anamorsin (UniProt IDs: Q6FI81). **(A)** Functional disorder profile generated by the D^2^P^2^ platform. **(B)** STRING-generated PPI network centered at anamorsin. **(C)** 3D structure modeled by AlphaFold.

Structurally, this protein can be split in several regions, namely, the N-terminal SAM-like domain (S-adenosyl-L-methionine-dependent methyltransferase domain) that does not bind S-adenosyl-L-methionine, but is required for correct assembly of the 2 Fe-S clusters (residues 6–172), a linker region (residues 173–224, two Fe-S binding sites (residues 237–251 and 274–288), and two Cx2C motifs (residues 274–277 and 285–288). Anamorsin contains a [2Fe-2S] cluster coordinated by C-terminally located cysteine residues 237, 246, 249, and 251 within the first Fe-S cluster-binding site and a [4Fe-4S] cluster coordinated by cysteine residues 274, 277, 285, and 288 within the second Fe-S binding site. The Cx2C motifs are required for the recognition by the mitochondrial CHCHD4/MIA40-GFER/ERV1 disulfide relay system, and when two disulfide bonds in the Cx2C motifs are formed through the dithiol/disulfide exchange reactions, anamorsin becomes effectively trapped in the mitochondrial intermembrane space (IMS) (Banci et al., 2011; Song et al., 2014), being the first identified Fe/S protein imported into the IMS (Banci et al., 2011). the N-terminal SAM-like domain is ordered, and its structure was solved by both X-ray crystallography [PDB ID: 4M7R; (Song et al., 2014)] and solution NMR spectroscopy (PDB IDs: 2LD4 (Banci et al., 2011) and 2YUI). Structural analysis of this domain revealed that although its structure closely resembles a typical SAM fold, it lacks one α-helix and one β-strand, making it incapable of conducting the S-adenosyl-L-methionine (AdoMet) dependent methyltransferase activity (Song et al., 2014). On the other hand, although the C-terminal domain of anamorsin contains a CIAPIN1 domain characterized by the presence of a highly conserved cysteine pattern, i.e., CX_5-14_CX_2_CXCX_n_CX_2_CX_7_CX_2_C and is able to bind 2 Fe-S clusters, it is mostly intrinsically disordered (Banci et al., 2011). This is evidenced by the results of limited proteolysis and solution NMR analysis, which indicated that the N-terminal part of anamorsin is well-folded, whereas the C-terminal part of this protein that includes the linker and CIAPIN1 domain, is largely unstructured and flexible (Banci et al., 2011). This notion is further supported Figure 10A showing that this C-terminal IDR contains 3 MoRFs (residues 115–123, 153–160, and 192–202) and multiple PTM sites. The STRING-generated PPI network of anamorsin contains 99 proteins involved in 860 interactions. In this network, with the average clustering coefficient of 0.777, the average node degree is 17.4, and the network contains significantly more than the 167 expected interactions (p-value <10^–16^), indicating that proteins in this network are at least partially biologically connected, as a group. In line with the data reported in Figure 10A, the 3D structure of human anamorsin generated by AlphaFold clearly indicated the complete lack of structure in the C-terminal half of this protein (see Figure 10C).

Based on the results of the FuzDrop analysis, human anamorsin can serve as a droplet-client, since its propensity for spontaneous LLPS is below 0.6 (p_LLPS_ = 0.4366), but it is predicted to have two DPRs (residues 209–219 and 259–282). It is yet to be found if the capability to be attracted to the biomolecular condensates is related to the functionality of anamorsin.

It is known that three isoforms of the human anamorsin are produced by alternative splicing, with isoform 2 missing residues 1–204 and isoform 3 being characterized by changing the region 53-66 (SAHKESSFDIILSG) to cysteine. No functional consequences of these sequence changes were reported as of yet. However, since the removal of the N-terminal region 1–204 in the isoform 2 eliminates all MoRFs, it is expected that interactability of human anamorsin will be noticeably affected. Furthermore, since N-terminal region of the canonical form of this protein includes SAM domain (residues 6–172), which is required for correct assembly of the 2 Fe-S clusters, the lack of this domain in isoform two is expected to affect the efficiency of the.

##### 3.2.3.2 DNA polymerase zeta catalytic subunit (UniProt IDs: O60673, PPIDR_PONDR VSL2_ = 61.18%)

The catalytic subunit of the DNA polymerase ζ complex [Pol ζ, the enzyme involved in mutagenic replication of damaged DNA (Morrison et al., 1989)], also known as POLZ or protein reversionless 3-like (REV3-like; REV3L; hREV3), has 3,130 residues and does not exhibit a proofreading function as it does not possess an intrinsic 3′-5′ exonuclease activity (Lee et al., 2014). REV3L is a B-family DNA polymerase (Burgers et al., 2001), catalytic activity of which is stimulated by the small REV7 subunit (Nelson et al., 1996). REV3 contains an iron-sulfur [4Fe-4S] cluster, required for the formation of active complex (Netz et al., 2011), is coordinated by the C-terminal cysteine residues 3,086, 3,089, 3,099, and 3,104, which form the CysB motif (residues 3,086–3104). The Pol ζ complex is dedicated to translesion synthesis (i.e., a DNA repair mechanism that allows cells to bypass DNA damage and continue DNA replication) and does not have any other known function in replication, recombination, or repair (Nelson et al., 1996; McGregor, 1999; Gan et al., 2008; Jansen et al., 2015; Martin and Wood, 2019).

**FIGURE 11:**
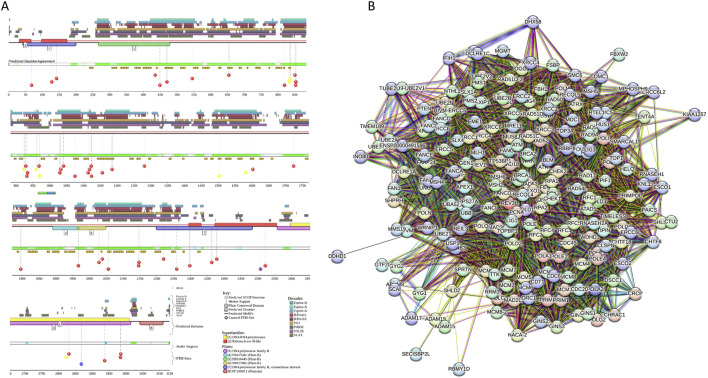
Functional disorder analysis of human REV3L (UniProt IDs: O60673). **(A)** Functional disorder profile generated by the D^2^P^2^ platform. **(B)** STRING-generated PPI network centered at REV3L.

Human REV3L contains several functional domains: The N-terminal domain (NTD, residues 1–333), the positively charged domain (PCD, residues 960–1200), the region mediating interaction with MAD2L2 (residues 1847–1899), the REV7-binding region (residues 1888–1943), the inactive 3′–5′ exonuclease domain (exo^−^, residues 2294–2532), the DNA polymerase domain (pol, residues 2558–3006), and the C-terminal domain (CTD, residues 3042–3109) (Makarova and Burgers, 2015). Figure 11A shows that human REV3L is predicted to contain high levels of intrinsic disorder, and its central region, spanning residues 200–2350, possesses 56 MoRFs and is heavily decorated with various PTMs. Importantly, the region mediating interaction with MAD2L2 includes two MoRFs (residues 1873–1882 and 1889–1910), and the REV7-binding region of REV3L contains 3 MoRFs (residues 1873–1882, 1889–1910, 1925–1934, and 1937–1957), suggesting that intrinsic disorder play a crucial role in the formation and stabilization of the corresponding complexes. High MoRF content defines binding promiscuity of human REV3L. This notion is supported by Figure 11B showing the STRING-generated PPI network centered at REV3L and containing 214 proteins linked by 8,552 interactions. This second largest network presented in this study is characterized by the average local clustering coefficient of 0.72 and has the highest average node degree of 79.9 (i.e., each on average, member of this network is engaged in almost 80 binary interactions). Obviously, this network contains significantly more interactions than expected (p-value <10^–16^). Unfortunately, due to its length, human RAV3 is not included in the AlphaFold Protein Structure Database (the minimum length is 16 amino acids, while the maximum is 2700 residues).

According to the FuzDrop analysis, human REV3L is expected to be a droplet-driver, as evidenced by its p_LLPS_ of 0.49998 and the presence of 30 DPRs (residues 265–300, 351–370, 393–414, 417–462, 465–549, 622–654, 684–745, 779–794, 810–894, 929–953, 959–974, 1016–1074, 1079–1095, 1103–1129, 1175–1237, 1247–1284, 1309–1320, 1372–1382, 1435–1455, 1511–1528, 1533–1597, 1606–1620, 1835–1920, 1957–1993, 2010–2059, 2064–2155, 2163–2187, 2204–2251, 2257–2277, 2305–3321, and 3012–3030) that cover a very significant part of its sequence. Although current literature does not contain information on the implementations of LLPS in the functionality of REV3L, it is tempting to speculate that the capability to form biomolecular condensates might contribute to the function of this important protein in the translesion DNA synthesis. This hypothesis is indirectly supported by the well-known involvement of biomolecular condensates in various processes associated with the DNA processing (Sabari, 2020; Sabari et al., 2020; Alavattam et al., 2021; Fijen and Rothenberg, 2021; Spegg and Altmeyer, 2021; Somasundaram et al., 2022; Pavlova et al., 2023; Zuo et al., 2023; Li et al., 2024; Nordenskiold et al., 2024; Shan et al., 2024; Valyaeva and Sheval, 2024).

It was demonstrated that human REV3L exists in isoforms, the canonical isoform one and alternatively spliced isoform 2, which is different from the canonical form by missing N-terminal residues 1-78 (Morelli et al., 1998). It was established that only the reference isoform, and not the alternate isoform, was able to produce protein *in vitro* and in cellular translation systems, suggesting that the alternative non-functional transcript isoform could help keeping low the cellular levels of the REV3L protein (Kawamura et al., 2001; Martin and Wood, 2019).

## Data Availability

The datasets presented in this study can be found in online repositories. The names of the repository/repositories and accession number(s) can be found in the article/Supplementary Material.
