# Supplementary material for: Flexible iron: disorder in the ironome brings order to protein structure and function
Source: Front Mol Biosci. 2025 May 30;12:1537164. doi: 10.3389/fmolb.2025.1537164 (PMC12162314; doi:10.3389/fmolb.2025.1537164)
Supplement: Supplementary file 1 [file Supplementaryfile1.docx]

**Supplementary Materials**

Flexible iron: Disorder in the ironome brings order to protein structure and function

Vladimir N. Uversky^1,2,*^ and Gloria C. Ferreira^1,3,4*^

^1^ Department of Molecular Medicine, Morsani College of Medicine, University of South Florida, Tampa, FL, USA

^2^ Byrd Alzheimer’s Center and Research Institute, Morsani College of Medicine, University of South Florida, Tampa, FL, USA

^3^ Department of Chemistry, College of Arts and Sciences, University of South Florida, Tampa, FL, USA

^4^ Global and Planetary Health, College of Public Health, University of South Florida, Tampa, FL, USA

**Supplementary Table S1**. Functional enrichment of the PPI networks centered at the entire human ironome, or human heme-, iron ion-, and iron-sulfur cluster-binding proteins

| **Protein set** | **ID** | **Description** | **Order of magnitude of the p-value** |
| --- | --- | --- | --- |
| **Human ironome** | **Biological Process (Gene Ontology)** | | |
|  | GO:0008152 | Metabolic process | -61 |
|  | GO:0044281 | Small molecule metabolic process | -49 |
|  | GO:0006082 | Organic acid metabolic process | -45 |
|  | GO:0019752 | Carboxylic acid metabolic process | -44 |
|  | GO:0043436 | Oxoacid metabolic process | -43 |
|  | **Molecular Function (Gene Ontology)** | | |
|  | GO:0016491 | Oxidoreductase activity | -215 |
|  | GO:0005506 | Iron ion binding | -169 |
|  | GO:0016705 | Oxidoreductase activity, acting on paired donors, with incorporation or reduction of molecular oxygen | -162 |
|  | GO:0020037 | Heme binding | -161 |
|  | GO:0046872 | Metal ion binding | -151 |
|  | **Cellular Component (Gene Ontology)** | | |
|  | GO:0005737 | Cytoplasm | -23 |
|  | GO:0005789 | Endoplasmic reticulum membrane | -23 |
|  | GO:0005739 | Mitochondrion | -23 |
|  | GO:0005783 | Endoplasmic reticulum | -22 |
|  | GO:0005622 | Intracellular anatomical structure | -18 |
|  | **Local Network Cluster (STRING)** | | |
|  | CL:11717 | Iron-sulfur cluster assembly, and Iron ion transport | -32 |
|  | CL:9385 | Steroid hormone biosynthesis, and Oxidation by cytochrome P450 | -32 |
|  | CL:5611 | A domain family that is part of the cupin metalloenzyme superfamily, and Syndromic X-linked intellectual disability Siderius type | -17 |
|  | CL:11797 | Iron-sulfur cluster assembly, and Essential protein Yae1, N-terminal | -16 |
|  | CL:11799 | Iron-sulfur cluster assembly | -15 |
|  | **KEGG Pathways** | | |
|  | hsa01100 | Metabolic pathways | -42 |
|  | hsa00590 | Arachidonic acid metabolism | -16 |
|  | hsa00830 | Retinol metabolism | -15 |
|  | hsa00140 | Steroid hormone biosynthesis | -10 |
|  | hsa05204 | Chemical carcinogenesis | -10 |
| **Human heme-binding proteins** | **Biological Process (Gene Ontology)** | | |
|  | GO:0120254 | Olefinic compound metabolic process | -41 |
|  | GO:0006082 | Organic acid metabolic process | -38 |
|  | GO:0044281 | Small molecule metabolic process | -37 |
|  | GO:0032787 | Monocarboxylic acid metabolic process | -36 |
|  | GO:0043436 | Oxoacid metabolic process | -36 |
|  | **Molecular Function (Gene Ontology)** | | |
|  | GO:0020037 | Heme binding | -204 |
|  | GO:0016491 | Oxidoreductase activity | -99 |
|  | GO:0004497 | Monooxygenase activity | -89 |
|  | GO:0016705 | Oxidoreductase activity, acting on paired donors, with incorporation or reduction of molecular oxygen | -84 |
|  | GO:0005506 | Iron ion binding | -80 |
|  | **Cellular Component (Gene Ontology)** | | |
|  | GO:0005789 | Endoplasmic reticulum membrane | -35 |
|  | GO:0031090 | Organelle membrane | -32 |
|  | GO:0005783 | Endoplasmic reticulum | -29 |
|  | GO:0012505 | Endomembrane system | -16 |
|  | GO:0005737 | Cytoplasm | -16 |
|  | **Local Network Cluster (STRING)** | | |
|  | CL:9385 | Steroid hormone biosynthesis, and Oxidation by cytochrome P450 | -43 |
|  | CL:9388 | Steroid hormone biosynthesis, and Arachidonic acid monooxygenase activity | -24 |
|  | CL:9392 | Retinol metabolism, and CYP2E1 reactions | -20 |
|  | CL:9393 | Retinol metabolism | -18 |
|  | CL:9394 | Retinoic acid 4-hydroxylase activity, and Arachidonic acid monooxygenase activity | -17 |
|  | **KEGG Pathways** | | |
|  | hsa01100 | Metabolic pathways | -27 |
|  | hsa00830 | Retinol metabolism | -18 |
|  | hsa00140 | Steroid hormone biosynthesis | -15 |
|  | hsa00590 | Arachidonic acid metabolism | -14 |
|  | hsa05204 | Chemical carcinogenesis | -13 |
| **Human iron ion-binding proteins** | **Biological Process (Gene Ontology)** | | |
|  | GO:0070988 | Demethylation | -46 |
|  | GO:0006482 | Protein demethylation | -38 |
|  | GO:0016577 | Histone demethylation | -37 |
|  | GO:0070076 | Histone lysine demethylation | -35 |
|  | GO:0018126 | Protein hydroxylation | -26 |
|  | **Molecular Function (Gene Ontology)** | | |
|  | GO:0051213 | Dioxygenase activity | -134 |
|  | GO:0016491 | Oxidoreductase activity | -107 |
|  | GO:0005506 | Iron ion binding | -101 |
|  | GO:0016705 | Oxidoreductase activity, acting on paired donors, with incorporation or reduction of molecular oxygen | -100 |
|  | GO:0016706 | 2-oxoglutarate-dependent dioxygenase activity | -95 |
|  | **Cellular Component (Gene Ontology)** | | |
|  | GO:0070013 | Intracellular organelle lumen | -5 |
|  | GO:0005622 | Intracellular anatomical structure | -5 |
|  | GO:0005737 | Cytoplasm | -3 |
|  | GO:0043231 | Intracellular membrane-bounded organelle | -3 |
|  | GO:0005955 | Calcineurin complex | -3 |
|  | **Local Network Cluster (STRING)** | | |
|  | CL:5611 | A domain family that is part of the cupin metalloenzyme superfamily, and Syndromic X-linked intellectual disability Siderius type | -25 |
|  | CL:5613 | Histone lysine demethylation, and Pyramidal neuron migration to cerebral cortex | -16 |
|  | CL:5615 | Histone H3-K9 demethylation, and Lysine-specific demethylase 6, GATA-like domain superfamily | -14 |
|  | CL:3223 | Mixed, incl. RNA N6-methyladenosine methyltransferase complex, and Alpha-ketoglutarate-dependent dioxygenase AlkB-like superfamily | -11 |
|  | CL:3277 | 2OG-Fe(II) oxygenase superfamily, and Cleavage furrow ingression | -11 |
|  | **KEGG Pathways** | | |
|  | hsa01100 | Metabolic pathways | -10 |
|  | hsa04216 | Ferroptosis | -6 |
|  | hsa04726 | Serotonergic synapse | -3 |
|  | hsa00790 | Folate biosynthesis | -3 |
|  | hsa04978 | Mineral absorption | -3 |
| **Human iron-sulfur-binding proteins** | **Biological Process (Gene Ontology)** | | |
|  | GO:0016226 | Iron-sulfur cluster assembly | -29 |
|  | GO:0044237 | Cellular metabolic process | -18 |
|  | GO:0006790 | Sulfur compound metabolic process | -16 |
|  | GO:0008152 | Metabolic process | -14 |
|  | GO:0097428 | Protein maturation by iron-sulfur cluster transfer | -11 |
|  | **Molecular Function (Gene Ontology)** | | |
|  | GO:0051536 | Iron-sulfur cluster binding | -129 |
|  | GO:0051539 | 4 iron, 4 sulfur cluster binding | -81 |
|  | GO:0051537 | 2 iron, 2 sulfur cluster binding | -38 |
|  | GO:0046872 | Metal ion binding | -28 |
|  | GO:0043167 | Ion binding | -21 |
|  | **Cellular Component (Gene Ontology)** | | |
|  | GO:0005739 | Mitochondrion | -22 |
|  | GO:0005759 | Mitochondrial matrix | -14 |
|  | GO:1990229 | Iron-sulfur cluster assembly complex | -13 |
|  | GO:0098803 | Respiratory chain complex | -07 |
|  | GO:0005746 | Mitochondrial respirasome | -07 |
|  | **Local Network Cluster (STRING)** | | |
|  | CL:11797 | Iron-sulfur cluster assembly, and Essential protein Yae1, N-terminal | -27 |
|  | CL:11799 | Iron-sulfur cluster assembly | -26 |
|  | CL:11717 | Iron-sulfur cluster assembly, and Iron ion transport | -26 |
|  | CL:11802 | Iron-sulfur cluster assembly complex, and Multiple mitochondrial dysfunctions syndrome 3 | -16 |
|  | CL:11818 | BolA-like protein, and Glutaredoxin, PICOT-like | -07 |
|  | **KEGG Pathways** | | |
|  | hsa00190 | Oxidative phosphorylation | -06 |
|  | hsa04932 | Non-alcoholic fatty liver disease | -06 |
|  | hsa03030 | DNA replication | -05 |
|  | hsa01100 | Metabolic pathways | -05 |
|  | hsa04714 | Thermogenesis | -05 |


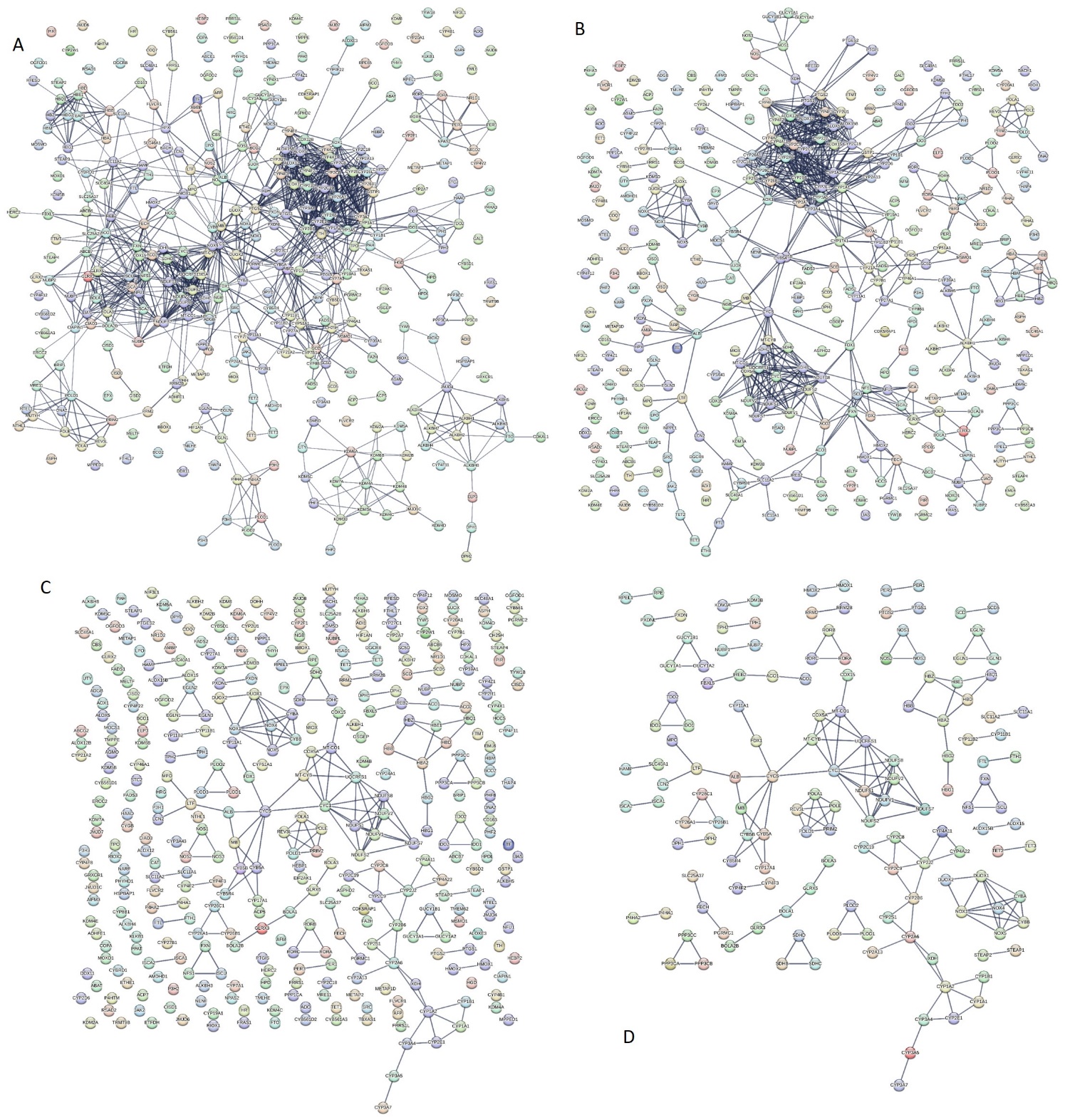


**Supplementary Figure S1**. **A**. Intra-set PPI network of human ironome generated using the confidence of the minimum required interaction score of 0.7 (high confidence). **B**. Intra-set PPI network of human ironome generated using the confidence of the minimum required interaction score of 0.9 (highest confidence). **C**. Intra-set PPI network of human ironome generated using only physical interactions at the highest confidence of 0.9. **D**. STRING-based PPI network generated for the 146 interacting members of the ironome from plot **C**. To generate this network, the highest confidence level of 0.9 was used and only physical sub-network, where the edges indicate that the proteins are part of a physical complex, was considered.


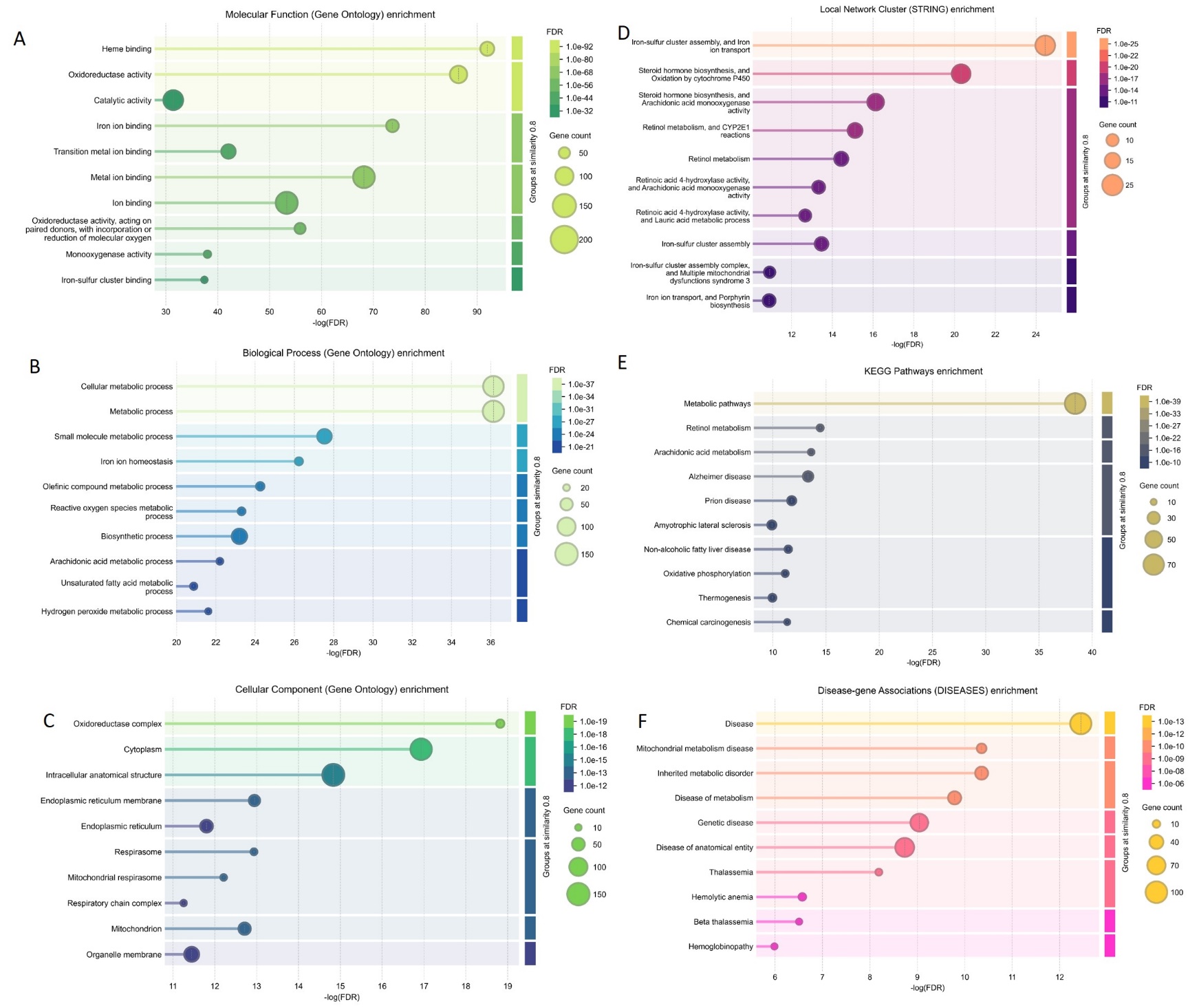


**Supplementary Figure S2**. STRING-based analysis of the functional enrichment of the members of the most stringent network generated by the 146 ironome members involved in physical interactions (see **Supplementary Figure 1D**). **A**. Molecular Functions (GO terms). **B**. Biological Processes (GO terms). **C**. Cellular Components (GO terms). **D**. Local network clusters (STRING), **E**. KEGG pathways (Ogata et al., 1999;Kanehisa et al., 2008;Kanehisa et al., 2010;Kanehisa et al., 2012;Kanehisa et al., 2025). **F**. Disease-gene associations. Corresponding data on the disease-gene associations are integrated to STRING from the DISEASES resource (Pletscher-Frankild et al., 2015;Grissa et al., 2022) accessible through a web interface at <http://diseases.jensenlab.org/>.

**References**

Grissa, D., Junge, A., Oprea, T.I., and Jensen, L.J. (2022). Diseases 2.0: a weekly updated database of disease-gene associations from text mining and data integration. *Database (Oxford)* 2022.

Kanehisa, M., Araki, M., Goto, S., Hattori, M., Hirakawa, M., Itoh, M., Katayama, T., Kawashima, S., Okuda, S., Tokimatsu, T., and Yamanishi, Y. (2008). KEGG for linking genomes to life and the environment. *Nucleic Acids Res* 36**,** D480-484.

Kanehisa, M., Furumichi, M., Sato, Y., Matsuura, Y., and Ishiguro-Watanabe, M. (2025). KEGG: biological systems database as a model of the real world. *Nucleic Acids Res* 53**,** D672-D677.

Kanehisa, M., Goto, S., Furumichi, M., Tanabe, M., and Hirakawa, M. (2010). KEGG for representation and analysis of molecular networks involving diseases and drugs. *Nucleic Acids Res* 38**,** D355-360.

Kanehisa, M., Goto, S., Sato, Y., Furumichi, M., and Tanabe, M. (2012). KEGG for integration and interpretation of large-scale molecular data sets. *Nucleic Acids Res* 40**,** D109-114.

Ogata, H., Goto, S., Sato, K., Fujibuchi, W., Bono, H., and Kanehisa, M. (1999). KEGG: Kyoto Encyclopedia of Genes and Genomes. *Nucleic Acids Res* 27**,** 29-34.

Pletscher-Frankild, S., Palleja, A., Tsafou, K., Binder, J.X., and Jensen, L.J. (2015). DISEASES: text mining and data integration of disease-gene associations. *Methods* 74**,** 83-89.
